# Demonstration of local adaptation in maize landraces by reciprocal transplantation

**DOI:** 10.1111/eva.13372

**Published:** 2022-04-14

**Authors:** Garrett M. Janzen, María Rocío Aguilar‐Rangel, Carolina Cíntora‐Martínez, Karla Azucena Blöcher‐Juárez, Eric González‐Segovia, Anthony J. Studer, Daniel E. Runcie, Sherry A. Flint‐Garcia, Rubén Rellán‐Álvarez, Ruairidh J. H. Sawers, Matthew B. Hufford

**Affiliations:** ^1^ 1177 Department of Ecology, Evolution, and Organismal Biology Iowa State University Ames Iowa USA; ^2^ Langebio Irapuato, Guanajuato Mexico; ^3^ Department of Crop Sciences University of Illinois Urbana‐Champaign Urbana Illinois USA; ^4^ Department of Plant Sciences University of California‐Davis Berkeley California USA; ^5^ Agricultural Research Service United States Department of Agriculture Columbia Missouri USA; ^6^ 14716 University of Missouri Columbia Missouri USA; ^7^ Present address: 1355 Department of Plant Biology University of Georgia Athens Georgia 30602 USA; ^8^ Present address: 6798 Molecular and Structural Biochemistry North Carolina State University 128 Polk Hall Raleigh North Carolina 27695‐7622 USA; ^9^ Present address: 145759 Department of Plant Science Pennsylvania State University University Park Pennsylvania 16802 USA

**Keywords:** highland adaptation, landrace, local adaptation, population genetics, reciprocal transplant, *Zea mays*

## Abstract

Populations are locally adapted when they exhibit higher fitness than foreign populations in their native habitat. Maize landrace adaptations to highland and lowland conditions are of interest to researchers and breeders. To determine the prevalence and strength of local adaptation in maize landraces, we performed a reciprocal transplant experiment across an elevational gradient in Mexico. We grew 120 landraces, grouped into four populations (Mexican Highland, Mexican Lowland, South American Highland, South American Lowland), in Mexican highland and lowland common gardens and collected phenotypes relevant to fitness and known highland‐adaptive traits such as anthocyanin pigmentation and macrohair density. 67k DArTseq markers were generated from field specimens to allow comparisons between phenotypic patterns and population genetic structure. We found phenotypic patterns consistent with local adaptation, though these patterns differ between the Mexican and South American populations. Quantitative trait differentiation (*Q*_ST_) was greater than neutral allele frequency differentiation (*F*_ST_) for many traits, signaling directional selection between pairs of populations. All populations exhibited higher fitness metric values when grown at their native elevation, and Mexican landraces had higher fitness than South American landraces when grown in these Mexican sites. As environmental distance between landraces’ native collection sites and common garden sites increased, fitness values dropped, suggesting landraces are adapted to environmental conditions at their natal sites. Correlations between fitness and anthocyanin pigmentation and macrohair traits were stronger in the highland site than the lowland site, supporting their status as highland‐adaptive. These results give substance to the long‐held presumption of local adaptation of New World maize landraces to elevation and other environmental variables across North and South America.

## 1 INTRODUCTION

Populations evolve adaptations to selective pressures imposed by biotic and abiotic environments. Over time, given sufficiently low genetic drift and gene flow, theory predicts that a population will adapt to the selective pressures of its local environment (Leimu & Fischer, 2008). In particular, populations are said to be locally adapted when they meet the “Foreign vs. Local” criterion of local adaptation, in which a local population exhibits higher fitness than foreign populations when grown in the same environment (Kawecki & Ebert, 2004).

Traditionally, attempts to identify and quantify local adaptation in natural populations have relied on common garden experiments (Clausen et al., 1940; Fraser et al., 2011; Savolainen et al., 2013; Turesson, 1922). Reciprocal transplant experiments are in many cases preferable to common garden experiments, as the scale, complexity, and variety of the environments of the included populations can be modeled more holistically, rather than being reduced to single or few environmental variables (Gibson et al., 2016; Kawecki & Ebert, 2004; Limpens et al., 2012; Savolainen et al., 2013). Exposing individuals from different populations to common environments can reveal that environments affect populations differently, a situation known as genotype‐by‐environment (*G* × *E*) interaction (Savolainen et al., 2013). Local adaptation is a type of *G* × *E* interaction in which a population has higher fitness in its native environment than any other non‐native population in that environment, illustrated by crossing fitness reaction norms in a reciprocal transplant experiment (Kawecki & Ebert, 2004; Savolainen et al., 2013).

Maize (*Zea mays* subsp. *mays*) is an extensively studied model system of high agronomic (Shiferaw et al., 2011), economic (Ranum et al., 2014; Shiferaw et al., 2011), cultural (Fernandez Suarez et al., 2013; Perales, 2016), and scientific (Dumas & Mogensen, 1993; Fedoroff, 2001; Stern et al., 2004) value. Maize was domesticated in the lowlands of the Balsas River Valley in Mexico from the teosinte taxon *Zea mays* subsp. *parviglumis* roughly 9000 years BP (Matsuoka et al., 2002). From there, maize was carried across North America and into South America as early as 6000 years BP (Bush et al., 1989; Grobman et al., 2012), north into the present‐day United States by about 4500 years BP (Merrill et al., 2009), and around the world as part of the Columbian exchange (Tenaillon & Charcosset, 2011; Van Heerwaarden et al., 2011). Presently, maize is grown across a greater range of elevations and latitudes than any other crop (Ruíz Corral et al., 2008; Shiferaw et al., 2011), encountering a broad range of temperature, precipitation, and soil types.

At locations along the historical range expansion of maize, farmers selected lines that were both suitable for growth in their local environment and desirable for human consumption and applications. Over generations of propagation and selection, this process formed varietal populations called landraces. These landraces are grown and maintained by smallholder farmers to the present day as dynamic, evolving populations (Dyer & Lopez‐Feldman, 2013; Mijangos‐Cortes et al., 2007) with low but significant gene flow between them (Ortega, 1995) (see Villa et al. (2005) for a review of the defining characteristics of landraces). Most of the arable land in Mexico is managed by subsistence farms that cultivate maize landraces (Bellon et al., 2018). Landraces are typically out‐yielded by modern hybrid maize (heterotic maize originating from controlled crosses of inbred parental lines) in industrial agricultural contexts, but in their home environments, landraces can and often do out‐perform commercial hybrids (Bellon et al., 2003, 2018; Mercer & Perales, 2018; Perales, 2016).

Maize landraces exhibit diverse morphological, physiological, and phenological characteristics, many of which co‐vary with climate, soil type and quality, and geography (Wellhausen et al., 1952). While farmers consciously select primarily for ear characteristics that are indirectly related to survival and reproduction (kernel filling, ear size, varietal consistency (Louette & Smale, 2000; Prasanna, 2010)), the environment selects for plant survival and reproduction (Cleveland & Soleri, 2007). The combination of these selective factors comprises the agroecosystem to which landraces adapt (Bracco et al., 2012; Villa et al., 2005).

Some of the most striking adaptations in maize landraces are in response to elevation (Eagles & Lothrop, 1994). Highland conditions present challenges for maize survival and productivity. At higher elevation, the atmosphere is thinner, leading to colder temperatures and less filtering of solar radiation. Marked phenotypic variation and genetic structure are correlated with elevation, though elevation itself may not be the causal agent (Dyer & Lopez‐Feldman, 2013). In at least some high‐elevation regions in Mexico, adaptations are hypothesized to be imparted via introgression from the maize wild relative *Zea mays* subsp. *mexicana* (hereafter “*mexicana*”), which is adapted to cool, dry highland conditions (Barnes et al., 2021; Hufford et al., 2012; Janzen et al., 2018; Lauter et al., 2004). Notable similarities between highland maize and *mexicana* include highly pigmented and pilose leaf sheaths (Doebley, 1984). Hufford et al. (2013) found that *mexicana* introgression into sympatric maize in Mexico overlapped chromosomal regions identified as QTL by Lauter et al. (2004) for pilosity and pigmentation (though other loci influence variance in these traits, e.g. *b1*, Selinger & Chandler, 2001). Leaf sheath anthocyanin pigmentation and pilosity have long been reported to help plants acquire and retain heat in cold environments (Doebley, 1984; Lauter et al., 2004; Schuepp, 1993). Anthocyanin pigmentation is plastically upregulated in response to increased light exposure (Vanderauwera et al., 2005) and cold temperatures (Christie et al., 1994; Hufford et al., 2013). Macrohair density is a plastic trait, associated with survival in cold temperatures (Hufford et al., 2013) and with maize grain yield in cold environments (Kaur et al., 1985). Dark red pigments absorb solar radiation, warming the plant. Pilosity increases surface friction, which decreases wind speed across the surface of the plant. This boundary layer around the plant reduces both heat loss and transpiration which can be advantageous in cool, dry regions (Chalker‐Scott, 1999; Schuepp, 1993).

There are multiple reasons to suspect that the nature of highland adaptations may differ significantly between landrace populations and between highland regions. First, highland adaptation seems to have evolved mostly independently in Mesoamerica and South America. Takuno et al. (2015) found that highland landraces in Mexico and South America were independently derived from lowland germplasm through selection on standing variation and *de novo* mutations, with little genomic evidence of convergent evolution. This hypothesis is supported by the absence of *mexicana* haplotypes (which are common in highland Mexican landraces and lacking in lowland Mexican landraces) in Andean highland landraces (Wang et al., 2017). Though more recent studies (Kistler et al., 2020; Wang et al., 2021) have found low but significant parallel highland adaptation between Mesoamerican and South American highland populations, the predominant pattern of highland adaptation remains independent. Second, selective pressures imparted by highland (and lowland) environments in Mesoamerica and South America are not identical. The strength and direction of correlations between elevation and climatic conditions can vary from one highland region to another. Precipitation and temperature correlate with elevation differently between Mexico and South America, and between lowland habitats west and east of highland ranges. In general, across Mexico, lowland conditions range from tropical to temperate, whereas highland conditions are cooler and drier (Medina Garcia et al., 1998). In South America, eastern lowlands neighbor the Amazon Basin, western coastal regions are arid, and southern highlands and lowlands become drier with increasing distance from the equatorial tropics (Sarmiento, 1975). The Andean rain shadow produces geographic regions with elevational gradients of cooler, moister highlands and hotter, dryer lowlands, across which indigenous farmers continue to cultivate maize and other crops (Brush, 1976). Because precipitation and temperature do not uniformly correlate with elevation, landraces that have evolved adaptations to high‐elevation bioclimatic conditions in South America may be ill‐suited for conditions found at the same elevation in Mexico.

Assessment of maize landrace local adaptation may prove valuable for modern maize breeders. The intense breeding programs that have developed modern inbred lines have drawn from limited germplasm and, through selection, have further reduced genetic diversity and capacity for adaptive plasticity (Gage et al., 2017). Reincorporation of landrace germplasm can restore key genetic variants that impart adaptations to challenging environments. Despite this potential, and despite a number of studies that report that local adaptation is pervasive among maize landraces (Bracco et al., 2012; Harlan, 1975; Navarro et al., 2017; Villa et al., 2005), research has not fully addressed whether maize landraces broadly do, in fact, exhibit reciprocal home‐site advantage, the definition of local adaptation. Landrace geographical extents have been shown to correspond to elevational and climatic factors (Aguirre‐Liguori et al., 2019; Arteaga et al., 2016; Ruíz Corral et al., 2008), supporting (but not demonstrating) local adaptation. Reciprocal transplant experiments set along an elevational gradient in the Mexican state of Chiapas (Mercer et al., 2008; Mercer & Perales, 2018) have shown that landraces local to that restricted area exhibit local adaptation. Taking a different approach, a recent study by Gates et al. (2019) found that landrace F1 hybrids (landrace individuals crossed with locally adapted testers) exhibit higher fitness and yield when grown at common garden sites closer to the native elevation of the landrace parent. This research identified promising candidate local adaptation loci, but sampling was restricted to Mexico and the signal of local adaptation was attenuated given the crossing scheme with adapted testers. The extent of local adaptation among maize landraces, therefore, has not been fully established.

To investigate the extent and degree of local adaptation between highland and lowland maize landraces, we conducted an elevational reciprocal transplant experiment. To test whether reportedly highland‐adaptive traits (macrohair and anthocyanin pigmentation) are truly adaptive in highland conditions, we calculated their correlation with fitness metrics at both common garden sites and compared them. Landraces’ fitness values were regressed to environmental distance between collection site and common garden site to determine if fitness values diminished when grown in environments more dissimilar from their native location. We also compared quantitative trait differentiation (*Q*_ST_) to neutral genetic differentiation between populations (*F*_ST_) to find traits under directional selection.

## 2 METHODS

### 2.1 Field experiment design

Landrace accessions from CIMMYT that met the following criteria were considered for inclusion in this experiment:

1. Accessions are present in the Seeds of Discovery (SeeDs) dataset (Pixley et al., 2017).
2. Accessions have latitude and longitude data from North or South America.
3. The elevation of the accession is below 1000 m or above 2000 m.

From eligible accessions, 30 pairs of highland and lowland accessions were chosen from both Mexico and South America (120 accessions total) such that both landraces of a pair were collected from the same 1‐degree of latitude bin, and all pairwise distances between accessions were greater than 50 km. These 120 samples were split into four populations (Mexican Highland, Mexican Lowland, South American Highland, South American Lowland, hereafter “Mex High,” “Mex Low,” “SA High,” and “SA Low”) with 30 accessions per population. We note that our provisional population designations are designed to reflect continental and elevational distinctions and not necessarily population genetic structure, and that we use the word “Mexican” to refer to the North American populations despite the fact that two of the accessions are from Guatemala. Passport data for the 120 landrace accessions are available in Table S1.

**FIGURE 1:**
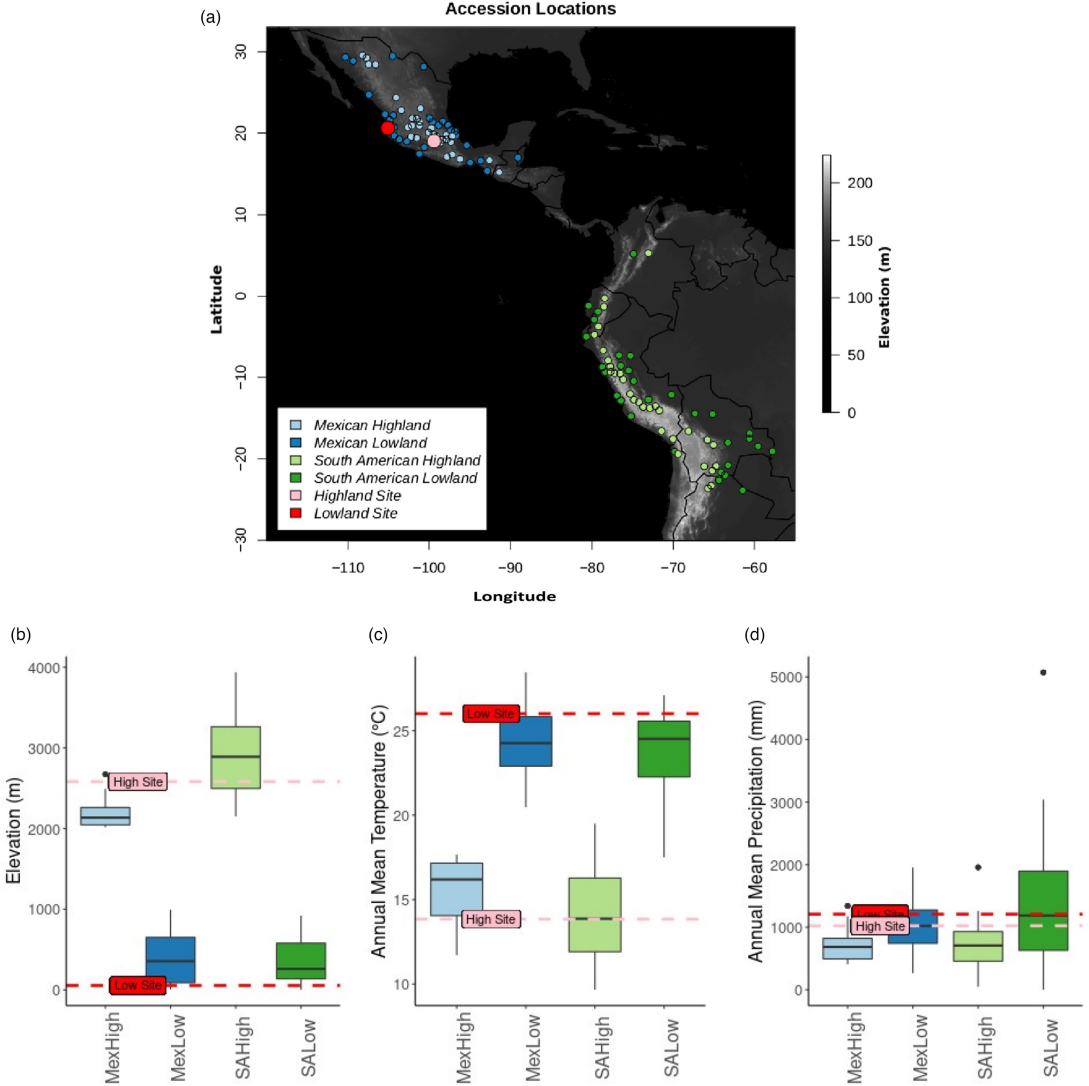
Geography and climate of 120 landraces and common garden sites. (a) Location of collection sites of landraces and common garden sites. Boxplots of topographical and environmental variables elevation (b), mean annual temperature (c), and mean annual precipitation (d) of landrace collection sizes. Red and pink dashed lines represent the values of the Lowland and Highland sites, respectively

The two common garden sites that comprise this reciprocal transplant are the Winter Services nursery site near Puerto Vallarta in the Pacific coastal lowlands (elevation 54 m) of Mexico (hereafter “Low Site”), and a CIMMYT field site near the town of Metepec in the highlands (elevation 2852 m) of the Mexican Central Plateau (hereafter “High Site”). Seed lines were regenerated at the field site in which they would be planted for one generation prior to the experiment to reduce seed storage and maternal effects. Best local practices for irrigation, fertilizer, and pest/weed control were used at both sites. The High Site field experiment was conducted in the summer of 2016. The Low Site field experiment was conducted in the winter of 2016, but virus damage led us to repeat the field experiment at the same site in the winter of 2017. Certain traits were collected from both years of the Low Site. A map of the field sites and geographical origin of each accession and boxplots summarizing the elevation, temperature, and precipitation distributions of these four populations are presented in Figure 1.

Each field was arranged in a complete block design with two blocks of 120 rows of 15 seeds of a landrace accession. Landraces from latitudinal pairs were planted in adjacent rows.

### 2.2 Phenotypic and genotypic data collection

Phenotypes (Table 1) were collected from the High Site and both years of the Low Site common gardens. Ear traits from the Low Site were collected from the 2016 season, but all other traits were taken from the 2017 growing season. Two healthy, representative plants from the interior of each row were selected and tagged. Individual plant phenotype data (plant height, ear height, ear number, tassel length, and tassel branch number) were collected from tagged plants. Other traits (stand count, ear‐producing stand count, barrenness, and flowering time) were collected at the row level. Days to anthesis and days to silking were recorded as the number of days until 50% of the row exhibited silk emergence or anther exertion on more than half of the main tassel spike, respectively. Anthesis‐silking interval was calculated as the difference in these two values.

**TABLE 1:** Names and descriptions of all collected phenotypes

Code	Trait Name	Unit of Measurement	Trait Description	Level
STD	Stand Count	Count	Number of plants surviving to sexual maturity	Row
PE	Ear‐Producing Stand Count	Count	Number of plants surviving to produce ears	Row
BRN	Barrenness	1‐(PE/STD)	Percent of plants in family that produce no ears	Row
DTA	Days to Anthesis	Count	Number of days between planting and 50% of plants exhibiting anthesis	Row
DTS	Days to Silking	Count	Number of days between planting and 50% of plants in the row silking	Row
ASI	Anthesis/Silking Interval	DTS‐DTA	Number of days between 50% silking and 50% anthesis	Row
PH	Plant Height	cm	Distance between the ground and the ligule of the flag leaf	Plant
EH	Ear Height	cm	Distance between the ground and the primary (top) ear‐bearing node	Plant
TL	Tassel Length	cm	Distance from top tip of the main spike to the attachment point of the bottom branch	Plant
TBN	Tassel Branch Number	Count	Number of tassel branches that attach to main spike	Plant
EN	Ear Number	Count	Number of seed‐producing ears produced	Plant
EW	Ear Weight	g	Mass of the primary ear	Plant
EL	Ear Length	cm	Length of the primary ear	Plant
KPR	Kernels per Row	Count	Number of kernels in a row on the primary ear	Plant
ED	Ear Diameter	cm	Diameter of the primary ear	Plant
δ^13^C	δ^13^C	[(*R*_Sample_/*R*_Standard_) – 1]*1000	Degree of inclusion of ^13^C in flag leaf tissue	Plant
FITplant	Agronomic Plant Fitness	PE/15 * √(EN) * EW	Adjusted plant fitness including yield metric	Plant
FITplantveg	Vegetative Plant Fitness	PE/15 * √(EN)	Adjusted plant fitness excluding yield metric	Plant
P_INTsolid	Pigment Intensity (Solid Pattern)	Visual 0–4 code scale	Visual assessment of the intensity of anthocyanin pigmentation	Plant
P_INTspot	Pigment Intensity (Spot Pattern)	Visual 0–4 code scale	Visual assessment of the intensity of anthocyanin pigmentation	Plant
P_EXTsolid	Pigment Extent (Solid Pattern)	Visual % code scale	Visual assessment of the extent of anthocyanin pigmentation from the ground up	Plant
P_EXTspot	Pigment Extent (Spot Pattern)	Visual % code scale	Visual assessment of the extent of anthocyanin pigmentation from the ground up	Plant
M_DENsolid	Macrohairs Density (Sheath)	Visual 0–4 code scale	Visual assessment of the density of sheath macrohairs of the second leaf from top	Plant
M_DENmarg	Macrohairs Density (Sheath Margin)	Visual 0–4 code scale	Visual assessment of the density of sheath margin macrohairs of the second leaf from top	Plant

Primary ears from tagged plants from the high site and the 2016 low site were returned to the lab to be photographed and processed for analysis. Total ear weight, ear length, ear diameter, and number of kernels per row were measured.

Methods for field visual assessment of anthocyanin pigmentation and macrohair were derived with modification from Lauter et al. (2004). Pigment was scored for pattern, intensity, and extent. The extent of leaf sheath anthocyanin pigmentation was visually scored as a percentage of the plant from ground level up (at 25% intervals). The intensity of leaf sheath pigmentation across the plant was visually scored on a scale of 0–4. Though all pigmentation patterns share some degree of genetic and environmental control, spots and banded/streaked patterns frequently co‐occur as an induced response to pathogenic stress (Selinger & Chandler, 1999), whereas uniform pigmentation (and leaf sheath macrohair expression) is shown to be inducible by highland conditions in some landraces (particularly those harboring introgressed QTL from *mexicana*). For these reasons, the “solid” pattern may have a stronger association with highland adaptation, and other patterns may represent stress responses to other biotic and/or abiotic factors. Plants were given the categorical qualitative label of either “banded,” “spotted,” “uniform,” or “no pattern” (either no pigment present or irregular pigment pattern). Plants with patterns of “banded” or “spotted” were binned into a “spot” group. Plants with pigment patterns “solid” and “no pattern” were binned into the group “solid.” When a plant exhibited multiple patterns, the highest‐priority category was selected (uniform, then banded, then spotted, then no pattern). Macrohair density on the second leaf sheath from the top of the plant was visually scored on a scale of 0–4. Pubescence along the leaf sheath and pubescence restricted to the sheath margin may be under different genetic control and may play different roles in highland adaptation. Therefore, plants were grouped by macrohair trait pattern (leaf sheath vs. leaf sheath margin).

Two adjusted fitness metrics were computed from the combination of several fitness traits (adapted from Mercer et al., 2008). Agronomic plant fitness (FITplant) incorporates the count of ear‐producing plants in the row (PE), the number of ears produced per plant (EN), and primary ear weight (EW). Ear‐producing stand count is divided by the number of seeds planted per row (15) to produce percent survival to sexual maturity, and ear number is square‐root transformed to account for diminishing yield returns of secondary, tertiary, and subsequent ears. To calculate adjusted fitness for plants that either did not produce ears by the time of harvest or were not harvested for collection of ear traits, a second plant fitness trait, vegetative plant fitness (FITplantveg), disregards ear weight from the equation. We calculate these adjusted fitness metrics thusly:

$$\text{FITplant}=\text{PE}/15*\sqrt{EN}*\text{EW}$$

$$\text{FITplantveg}=\text{PE}/15*\sqrt{EN}.$$

Flag leaves from tagged plants from High and 2016 Low Sites were collected for Carbon isotope discrimination analysis, which was carried out at the University of Illinois (Twohey et al., 2019). Carbon isotopic composition *δ* ^13^C was calculated in reference to the international standard, Vienna Pee Dee Belemnite. The equation for *δ* ^13^C (Schwarcz & Schoeninger, 1991) is as follows:

$$\delta^{13}\text{C =}\;\left\{\left[\frac{\left({}^{13}C_{\text{Sample}}:{}^{12}C_{\text{Sample}}\right)}{\left({}^{12}C_{\text{Standard}}:{}^{12}C_{\text{Standard}}\right)}\right]‐1\right\}*1000.$$

Leaf tissue samples were collected from a subset of 92 landraces in both High and Low Sites. DNA was extracted with the CTAB method (Doyle & Doyle, 1987) and sent to SAGA (Genetic Analysis Service for Agriculture) at CIMMYT for DArTseq genotyping (Wenzl et al., 2004), a reduced‐representation re‐sequencing technique designed and optimized for the complex genomes of maize and wheat. Following the standard DArTseq protocol, over 67,000 DArTseq SNP markers were generated.

### 2.3 Statistical analyses

#### 2.3.1 Population structure

Axes of population structure were estimated from SNP data with principal components analysis (R package KRIS, Chaichoompu et al., 2018).

To determine the degree to which our predefined continent/elevation populations conform to population genetic structure, we estimated the number of ancestral populations (*K*) and conducted admixture analysis. To estimate admixture coefficients from the genotypic data, we used sparse non‐negative matrix factorization (sNMF, R package LEA, Frichot & François, 2015), an algorithm similar to Bayesian clustering algorithms like STRUCTURE (Pritchard et al., 2000). sNMF was run assuming *K* values 2–9 with 50 repetitions per *K* value. The value of *K* was then selected via the cross‐entropy criterion. The visualization of the interpolation of admixture coefficients across geographic space was performed with TESS3 (R package tess3r, Caye et al., 2016).

#### 2.3.2 Phenotype:Phenotype correlations

Principal Components Analysis (function prcomp, R package stats, R Core Team, 2019) was used to study the relatedness between phenotypic patterns. Data were normalized via centering and scaling. Yield traits and *δ* ^13^C were available only from the second year of the Low Site and were therefore excluded from PCA.

We calculated Pearson correlations between phenotypic traits on a garden‐specific basis to identify how trait:trait correlations are affected by environmental differences between the two common gardens. *δ* ^13^C was excluded from this analysis due to the limited number of plants selected for measuring this trait. Correlation significance was based on a *t*‐test of Fisher's *z*‐transformation of Pearson's *r* coefficients (function cor.test, R package stats, R Core Team, 2019).

To determine the elevation‐dependent fitness consequences of putatively highland‐adaptive traits, we calculated Pearson correlations between fitness (FITplantveg) and traits previously identified and frequently reported as highland adaptive (Doebley, 1984; Eagles & Lothrop, 1994). FITplantveg was used rather than FITplant because FITplantveg had more complete data. These correlations were determined independently for each common garden site, and correlation significance was determined as above. Additionally, the magnitude and direction of differences in fitness/highland‐adaptive trait correlation coefficients between sites were taken as evidence of the trait's adaptive role at high or low elevation. Two classes of pigment and macrohair patterns (either “solid”/“spotted” or “solid”/“margin”, respectively) were also considered separately.

#### 2.3.3 G×E interactions

We used a linear mixed‐effects model (R package lme4, Bates et al., 2014) to test for phenotypic differences between landraces from each of the four populations and to test how these differences changed between the two common gardens. The full model was specified as:

$$\begin{array}{c} \text{TRAIT}∼\text{GARDEN}*\text{CONTINENT}*\text{ELEVATION}+\\ \text{BLOCK}:\text{GARDEN}+(1|\text{LATITUDE})+(1|\text{LATITUDE}:\text{GARDEN)}. \end{array}$$

This formula calls as fixed effects GARDEN (Low Site or High Site), CONTINENT (Mexico or South America), ELEVATION (High or Low), all interaction combinations therein, BLOCK nested in GARDEN, and calls as random effects with random intercept accession LATITUDE and LATITUDE/GARDEN interaction. The significance of specific treatment effects was evaluated using the lmerTest (Kuznetsova et al., 2017) and lsmeans (Lenth, 2012) R packages, and Bonferroni correction (Bonferroni, 1936) was used to account for multiple comparisons (for tests of each trait within each contrast).

We compared each population's phenotypes between field sites (to quantify *G*×*E* interactions), between highland and lowland populations from the same continent within each field site (to quantify highland‐lowland adaptation), and between Mexican and South American populations from the same elevation within each field site (to quantify adaptation to continent‐specific factors).

#### 2.3.4 *Q*_ST_/*F*_ST_ comparison

Quantitative trait divergence (*Q*_ST_) was contrasted to the distribution of *F*_ST_ for neutral genetic markers (Whitlock, 2008). For traits in which *Q*_ST_ > *F*_ST_, trait divergence is greater than neutral expectations, which may be caused by directional selection (Leinonen et al., 2013).

A linear mixed effects model was used to partition phenotypic variance between population, landrace accession line, and garden/block:

TRAIT∼1+(1|POPULATION)+(1|LINE)+(1|GARDEN/BLOCK)

Pairwise *F*_ST_ was calculated with the R function *F*_ST_.each.snp.hudson (R package dartR, Gruber et al., 2018). Within‐population and between‐population variances were calculated with the R function VarCorr (R package lme4, Bates et al., 2014), and were used to calculate *Q*_ST_ following the equation below:

$$Q_{\text{ST}}=\sigma_{\text{GB}}^{2}/\left(\sigma_{\text{GB}}^{2}+2\sigma_{\text{GW}}^{2}\right)$$

in which *σ*_GB_^2^ and *σ*_GW_^2^ are the between‐ and within‐population genetic variance components, respectively (Leinonen et al., 2013). Population contrasts of interest were all highland vs. all lowland, all Mexican vs. all South American, Mexican Highland vs. Mexican Lowland, and South American Highland vs. South American Lowland. *Q*_ST_ values were considered significantly high if they were more than two standard deviations above the mean *F*_ST_.

#### 2.3.5 Environmental distance effects

Bioclimatic environmental values were extracted from 30 s (~1 km^2^) resolution global GeoTiff files downloaded from WorldClim 2.1 (Fick & Hijmans, 2017). Elevation values were included in the passport data for the landrace accessions, and therefore did not need to be extracted.

In keeping with methods employed by Gates et al. (2019), we regressed fitness residuals to environmental distance using a quadratic model to detect diminishing fitness across greater environmental distance. First, environmental distance was calculated for each environmental variable as the value of the environmental variable at the common garden site minus the value of the variable at the landrace's accession origin site (*DISTANCE* = *Value_GARDEN_* −*Value_ORIGIN_*). A negative environmental distance value signifies that the environmental value at the landrace's origin location was lower than that of the common garden site. We then regressed vegetative fitness (FITplantveg) against environmental distance with a linear model (*FITplantveg* ~ *DISTANCE*) for each environmental variable. To remove the effect of unequal mean fitness between common garden sites, we used the residuals of this linear model (hereafter fitness residuals, *e*). Next, to test if fitness residuals decreased with increasing (more positive or more negative) environmental distances, fitness residuals were fit with a quadratic model (*e* ~ *DISTANCE* +*DISTANCE* ^2^). The relationship between the fitness residuals and the quadratic coefficient is quantified with a *t*‐value with an associated *p*‐value to denote the probability of no relationship. A significant *p*‐value therefore indicates that the fitness residuals follow a quadratic (parabolic) trend more closely than would be expected by chance. The proportion of fitness residual variance explained by the quadratic model is expressed with *R* ^2^.

## 3 RESULTS

### 3.1 Population structure

**FIGURE 2:**
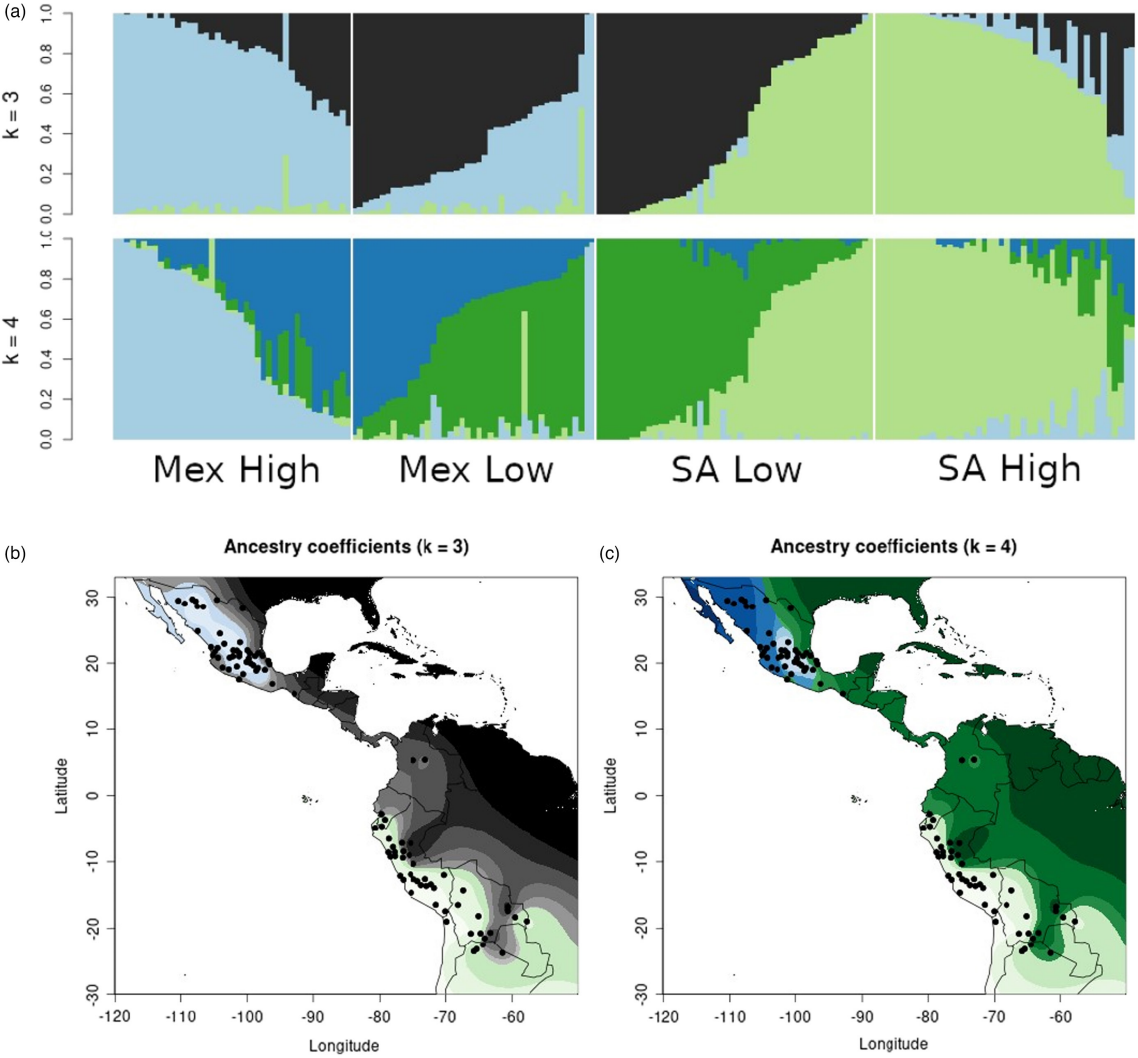
Population structure of the 120 landrace accessions. Ancestry coefficients were calculated by sNMF at optimal *K* values 3 and 4 and plotted in STRUCTURE‐like bar charts (a). Vertical lines represent individual plants, which are binned into pre‐defined populations and sorted by admixture coefficient value. These ancestry coefficients were interpolated over a geographic map of the study region (b, c)

Genetic similarity between genotyped individuals based on SNP data was first estimated with principal components analysis (Figure S1). PC1 (24.7%) primarily separates Mexican from South American populations, and PC2 (14.4%) primarily separates highland from lowland populations. sNMF afforded greater clarity into patterns of population structure. The cross‐entropy criterion identified an optimal *K* = 3 ancestral populations (Figure S2). We also considered *K* = 4 to permit comparison between our four continent/elevation populations and those identified by sNMF. STRUCTURE‐like bar charts show admixture between ancestral populations identified by sNMF at both *K* = 3 and *K* = 4 (Figure 2a). At *K* = 3, Mex Low and SA Low are combined into one group, indicating that these two populations are the least differentiated. At *K* = 4, recognizable ancestry groups are clear, with admixture mostly between adjacent populations (Mex High and Mex Low, Mex Low and SA Low, SA Low and SA High). Admixture coefficients for both *K* values are plotted across geographic space to demonstrate their distributions (Figure 2b,c).

### 3.2 Phenotypic summary

Broad‐scale phenotypic trait patterns were first ascertained with principal components analysis. Traits with low missing data between the three gardens (the High Site and both years of the Low Site) were used to perform PCA. The first two components distinguish individuals from the High Site from both plantings of the Low Site (Figure S3a). The two years of the Low Site share a higher degree of feature space overlap than either shares with the High Site. High values of anthocyanin intensity, anthocyanin extent, and days to anthesis and silking characterize plants from the High Site. High values of several fitness‐related traits distinguish the Low Site 2017 from the Low Site 2016 and the High Site. Populations overlap strongly in the first two components of PC space (Figure S3b).

Garden‐specific Pearson correlations of phenotypic traits were calculated and plotted (Figure S4). Black squares demark the top five clusters of correlated traits within each garden site, and asterisks denote level of significance.

### 3.3 Local adaptation

#### 3.3.1 Highland adaptation traits

**FIGURE 3:**
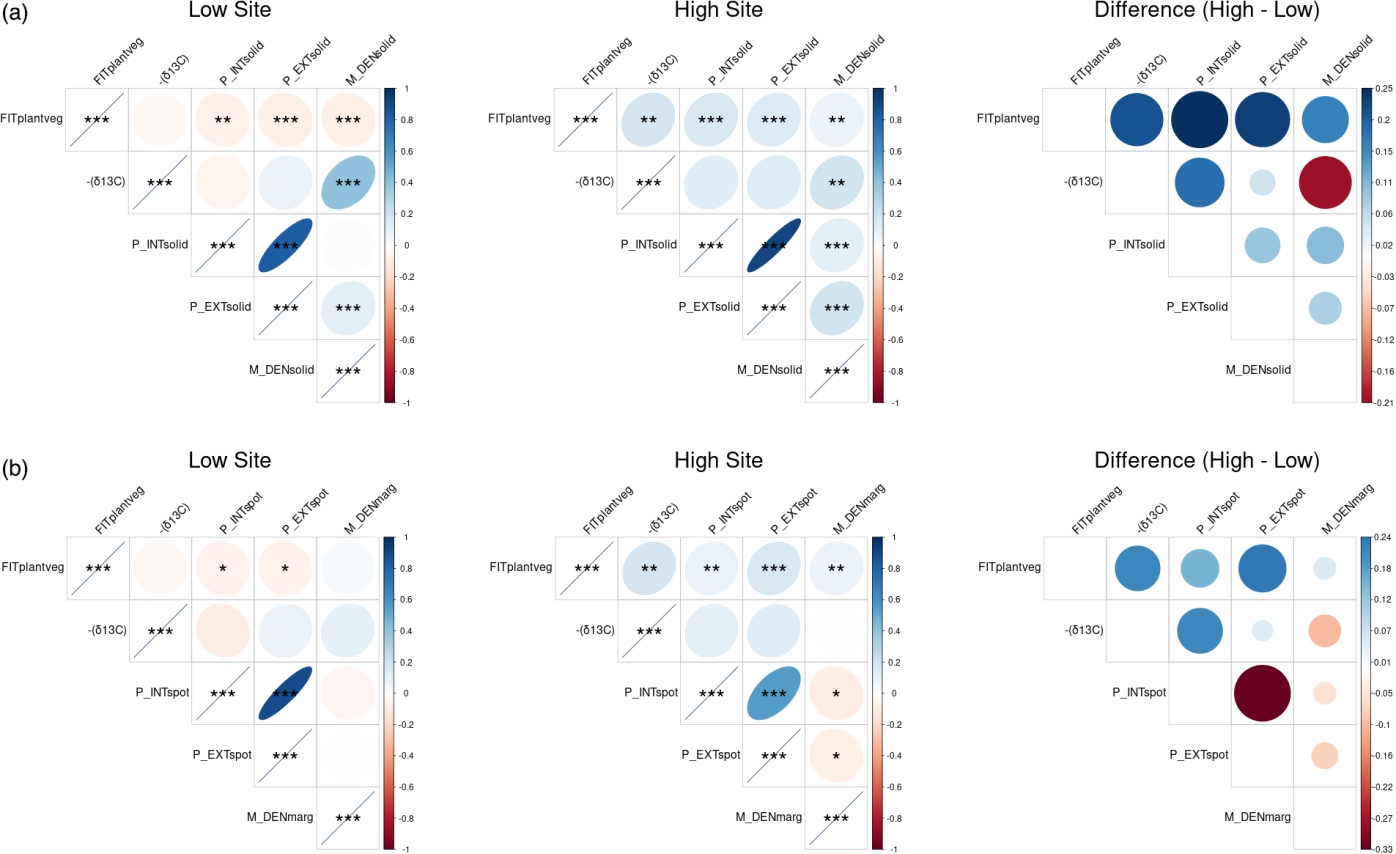
Pearson correlation between plant vegetative fitness (FITplantveg), *δ* ^13^C, and traits putatively related to highland adaptation. The inverse of *δ* ^13^C is used in this case so that more positive values can be more directly associated with water use efficiency. (a) Solid‐pattern anthocyanin pigmentation intensity (P_INTsolid) and extent (P_EXTsolid), and leaf sheath macrohair density (M_DENsolid). (b) Irregular‐pattern anthocyanin pigmentation intensity (P_INTspot) and extent (P_EXTspot), and leaf sheath margin macrohair density (M_DENmarg). For each subfigure, panels 1 and 2 show correlations within the Low Site and the High Site, blue shapes indicate positive correlation, red shapes indicate negative correlation, color intensity and shape size indicate strength of correlation, and asterisks indicate statistical significance (*p*‐value thresholds = 0.05, 0.01, 0.001). Panel 3 shows the between‐garden difference in correlation value for each pairwise correlation (positive/blue values indicate more positive correlations in the highland site than in the lowland site)

Pearson correlation values between fitness, the inverse of *δ* ^13^C (hereafter −*δ* ^13^C), pigment traits, and macrohair traits vary between both common gardens (Figure 3). Differential patterns in pigment and macrohair traits seem to influence fitness correlations.

In the Low Site, plant vegetative fitness (FITplantveg) is strongly negatively correlated with solid‐pattern anthocyanin pigmentation intensity (P_INTsolid) and extent (P_EXTsolid) and leaf sheath macrohair density (M_DENsolid). In the High Site, however, these correlations become strongly positive, suggesting that these traits are more advantageous in the High Site than the Low Site. A similar (but weaker) pattern is seen with fitness correlations with spot‐pattern anthocyanin pigmentation intensity (P_INTspot) and extent (P_EXTspot) and leaf margin macrohair density (M_DENmarg). This suggests that expression of the alternate patterns of these traits is advantageous in the High Site, but less so than for the solid‐pattern traits.

−*δ* ^13^C can be thought of as roughly analogous to water use efficiency (WUE). This trait is more correlated with fitness in the High Site than in the Low Site, suggesting that WUE is more advantageous in the highland environment. −*δ* ^13^C has no significant correlations with anthocyanin pigmentation intensity or extent, regardless of pattern or garden site, nor with leaf margin macrohair density, but it does correlate positively with leaf sheath macrohair density in both sites (though, notably, this correlation is more significant in the Low Site). This may suggest that leaf sheath macrohair density may contribute towards WUE.

#### 3.3.2 Population mean reaction norms

Reaction norms describe phenotypic trait values of genotypes (in this case, landrace populations) at different environments (common garden sites). Nonparallel reaction norms indicate that populations respond to environments differently, a pattern known as genotype‐by‐environment (*G* × *E*) interaction. When local populations have fitness trait values higher than the fitness trait values of non‐local populations, resulting in crossed reaction norms, this is known as local adaptation.

**FIGURE 4:**
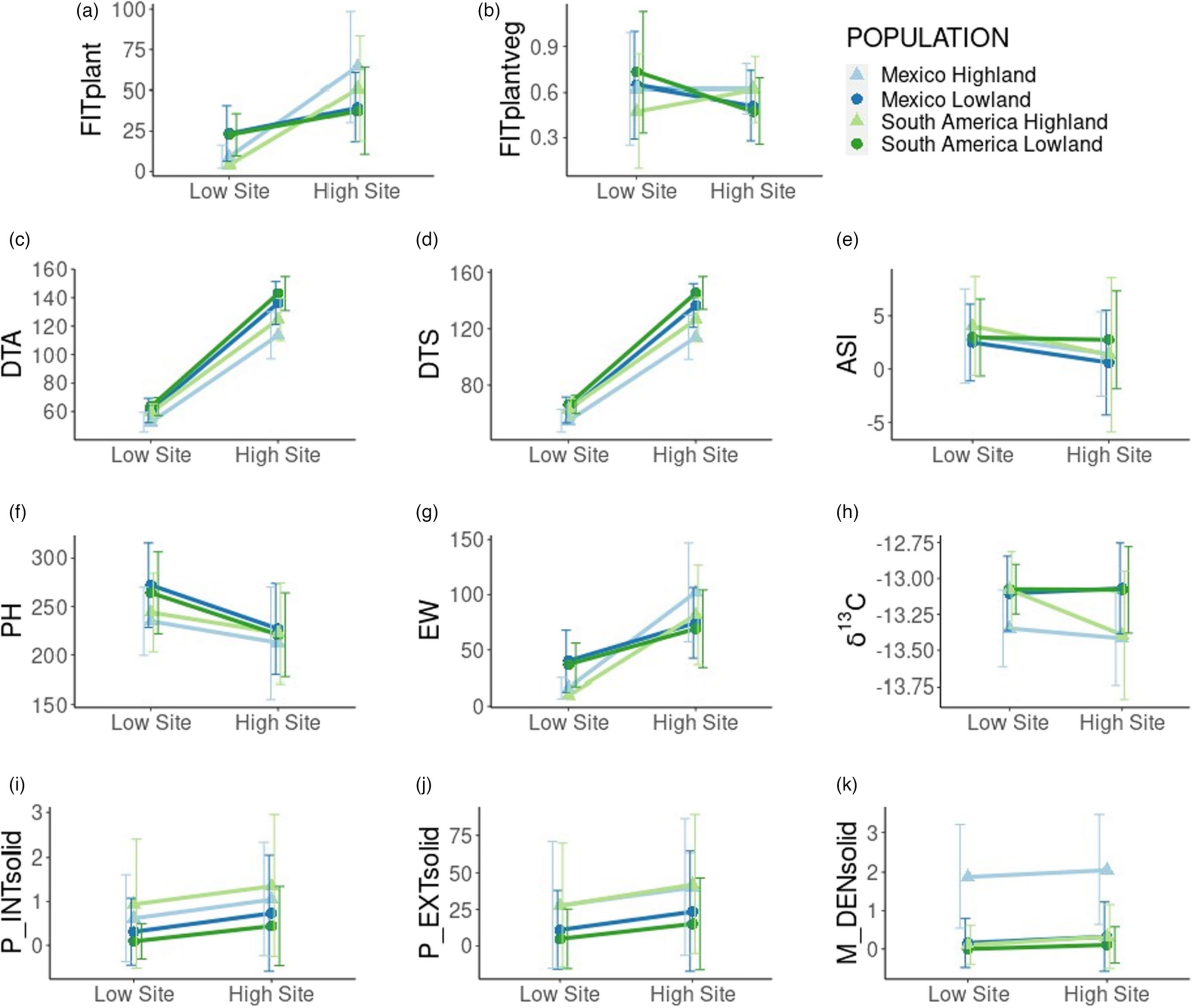
Reaction norms for selected measured phenotypic traits agronomic plant fitness (FITplant, a), vegetative plant fitness (FITplantveg, b), days to anthesis (DTA, c), days to silking (DTS, d), anthesis‐silking interval (ASI, e), plant height (PH, f), ear weight (EW, g), *δ* ^13^C (*δ* ^13^C, h), solid‐pattern anthocyanin pigmentation intensity (P_INTsolid, i) and extent (P_EXTsolid, j), and leaf sheath macrohair density (M_DENsolid, k). Error bars denote standard deviation

Reaction norms for all traits are available in Figure S5, and a selection of these plots are available in Figure 4. A full report of the statistical significance of each contrast is provided in Tables 2 and 3. Both agronomic fitness (FITplant, Figure 4a) and vegetative fitness (FITplantveg, Figure 4b) showed strong patterns of home‐site advantage. In the High Site, high populations had higher agronomic fitness (*t*‐ratio = 11.799, *p *< 0.0001) and vegetative fitness (*t*‐ratio = 10.153, *p *< 0.0001) than low populations. In the Low Site, Low populations had higher vegetative fitness than High populations (*t*‐ratio = −7.117, *p *< 0.0001), though differences in agronomic fitness were less clear (*t*‐ratio = −1.762, *p* = 0.079). In all but one contrast, Mex populations had higher fitness than SA populations from the same elevation, though the significance of these differences is generally less than those of High/Low population contrasts. Agronomic fitness was higher in Mex High than in SA High in the High Site (*t*‐ratio = 5.417, *p *< 0.0001), and vegetative plant fitness was higher in Mex High than in SA High in the Low Site (*t*‐ratio = 4.900, *p *< 0.0001). However, vegetative plant fitness was lower in Mex Low than in SA Low (*t*‐ratio = −4.138, *p *< 0.0001) at the Low Site.

**TABLE 2:** Reaction norm *t*‐ratios

Garden	H/L Site	H/L Site	H/L Site	H/L Site	Low Site	Low Site	High Site	High Site	Low Site	Low Site	High Site	High Site
Continent	Mex	Mex	SA	SA	Mex/SA	Mex/SA	Mex/SA	Mex/SA	Mex	SA	Mex	SA
Elevation	High Pop	Low Pop	High Pop	Low Pop	High Pop	Low Pop	High Pop	Low Pop	H/L Pop	H/L Pop	H/L Pop	H/L Pop
FITplant	6.1125	3.3423	3.6216	2.6360	0.4080	0.2344	5.4171	0.4077	−1.3247	−1.2357	11.0780	5.7121
FITplantveg	2.4790	−0.9804	6.2709	−4.5484	4.8997	−4.1384	−0.4623	1.0394	−0.4844	−9.5586	6.4246	7.9262
STD	−5.3756	−9.3367	−7.8128	−14.9247	0.1002	−3.2542	1.5785	0.6101	−0.3010	−3.7004	2.3377	1.4210
PE	0.7723	−1.8482	2.8144	−4.1440	2.7617	−1.4433	1.3215	0.2282	0.8923	−3.3841	2.8361	1.8222
BRN	−1.1640	4.0683	−4.9270	2.4451	−4.0977	−0.8449	−0.5251	0.8254	1.2953	4.7032	−3.8702	−2.6620
DTA	29.6693	36.0169	32.7339	40.5317	−2.4027	−0.7745	−3.5142	−1.5387	−2.9643	−1.3710	−7.8249	−6.2827
DTS	30.3550	37.4582	33.0647	42.3751	−2.9695	−0.9967	−4.0579	−2.1328	−2.7057	−0.7444	−7.7712	−6.2611
ASI	−1.5595	−2.1965	−2.8135	−0.3332	−1.0014	−0.3451	0.0630	−2.2237	0.3791	1.0884	1.0250	−1.2913
PH	−2.4253	−5.6881	−2.3834	−5.0938	−0.9649	0.9659	−1.0950	0.5075	−3.3913	−1.5368	−1.3876	0.1957
EH	−0.3349	−2.6738	−3.1234	−2.8805	−2.5966	−0.2448	−1.1322	−0.2045	−3.4023	−1.0618	−2.1902	−1.3329
TL	−0.3748	−4.5493	−1.0132	−3.9481	−1.2782	0.3102	−0.9539	−0.4485	−3.0755	−1.6442	−0.1852	0.3218
TBN	−1.1173	−0.8036	−1.6751	−6.3568	−6.6390	−6.8510	−6.7148	−3.7523	−4.4176	−4.8626	−4.8456	−2.1117
EN	0.9688	1.6559	5.3970	1.7051	2.2344	−0.0538	−1.7088	−0.0630	0.8728	−1.4563	0.3058	2.0244
EW	6.8717	5.1858	3.8544	4.1746	0.2458	0.5919	6.1063	1.1231	−1.6966	−1.1467	9.1690	3.9633
EL	9.0527	3.5386	4.5299	3.9804	−1.3502	0.2708	1.9446	−0.5138	−6.4089	−2.9580	1.4053	−1.0438
KPR	3.7061	−6.0551	−0.5150	−2.7173	−1.1965	4.3278	6.5842	0.9395	−7.3278	−1.9882	1.9880	−3.4545
ED	13.4858	10.5086	13.7368	11.1622	2.4820	−0.4043	−3.1635	−2.2981	−3.8080	−5.7584	7.3276	7.6050
δ^13^C	−1.2743	0.2486	−4.3935	0.0547	−2.9274	−0.1003	−0.5420	0.0928	−2.7349	−0.0530	−5.0014	−5.0299
P_INTsolid	2.9732	2.2258	2.2588	2.2805	−2.0258	0.8985	−1.5251	0.9918	0.5985	3.6465	1.3712	4.0111
P_INTspot	4.8977	4.3411	3.3825	3.6824	−3.6488	−1.3506	−2.4661	−0.6210	−0.1697	2.4569	0.7089	2.7436
P_EXTsolid	2.8202	2.1741	2.6008	2.3123	−0.9545	0.8467	−0.7485	0.8733	1.1635	3.0702	1.9103	3.6512
P_EXTspot	6.1588	5.3073	5.3158	7.4200	−1.7798	−0.0127	−1.1406	−1.5102	0.9735	2.9143	2.2733	2.1094
M_DENsolid	0.8312	1.2038	1.6496	0.8252	8.4631	0.9575	8.4936	1.1745	8.1827	0.7004	8.3710	1.1267
M_DENmarg	−1.8743	−1.1438	−2.8539	−1.6256	1.3160	1.2018	1.3782	1.7602	1.0142	0.7862	−0.3084	−0.4601

Note

*t*‐ratios are the estimated effect size of the contrast divided by standard error. Each *t*‐ratio corresponds to a phenotypic contrast between two groups that differ in level of Garden (H/L Site), Continent (Mex/SA), or Population (H/L Pop), while other levels are held constant.

**TABLE 3:** *p*‐Values of reaction norm *t*‐ratios

Garden	H/L Site	H/L Site	H/L Site	H/L Site	Low Site	Low Site	High Site	High Site	Low Site	Low Site	High Site	High Site
Continent	Mex	Mex	SA	SA	Mex/SA	Mex/SA	Mex/SA	Mex/SA	Mex	SA	Mex	SA
Elevation	High Pop	Low Pop	High Pop	Low Pop	High Pop	Low Pop	High Pop	Low Pop	H/L Pop	H/L Pop	H/L Pop	H/L Pop
FITplant	**0.0000**	**0.0012**	**0.0003**	**0.0092**	0.6834	0.8147	**0.0000**	0.6836	0.1858	0.2170	**0.0000**	**0.0000**
FITplantveg	0.0158	0.3303	**0.0000**	**0.0000**	**0.0000**	**0.0000**	0.6440	0.2988	0.6281	**0.0000**	**0.0000**	**0.0000**
STD	**0.0000**	**0.0000**	**0.0000**	**0.0000**	0.9203	**0.0014**	0.1162	0.5425	0.7638	**0.0003**	0.0205	0.1570
PE	0.4416	0.0672	**0.0058**	**0.0001**	**0.0063**	0.1506	0.1880	0.8198	0.3734	**0.0009**	**0.0051**	0.0700
BRN	0.2470	**0.0001**	**0.0000**	0.0161	**0.0001**	0.3991	0.6000	0.4101	0.1966	**0.0000**	**0.0001**	**0.0083**
DTA	**0.0000**	**0.0000**	**0.0000**	**0.0000**	0.0174	0.4397	**0.0006**	0.1256	**0.0035**	0.1722	**0.0000**	**0.0000**
DTS	**0.0000**	**0.0000**	**0.0000**	**0.0000**	**0.0034**	0.3204	**0.0001**	0.0344	**0.0076**	0.4577	**0.0000**	**0.0000**
ASI	0.1219	0.0301	**0.0058**	0.7396	0.3176	0.7303	0.9498	0.0274	0.7049	0.2775	0.3067	0.1983
PH	0.0169	**0.0000**	0.0188	**0.0000**	0.3359	0.3354	0.2752	0.6125	**0.0009**	0.1261	0.1672	0.8451
EH	0.7384	**0.0086**	**0.0023**	**0.0048**	**0.0103**	0.8070	0.2595	0.8382	**0.0009**	0.2899	0.0301	0.1847
TL	0.7086	**0.0000**	0.3132	**0.0001**	0.2027	0.7567	0.3417	0.6544	**0.0024**	0.1017	0.8533	0.7480
TBN	0.2665	0.4233	0.0968	**0.0000**	**0.0000**	**0.0000**	**0.0000**	**0.0002**	**0.0000**	**0.0000**	**0.0000**	0.0364
EN	0.3347	0.1004	**0.0000**	0.0908	0.0264	0.9572	0.0891	0.9498	0.3836	0.1466	0.7601	0.0443
EW	**0.0000**	**0.0000**	**0.0001**	**0.0000**	0.8059	0.5541	**0.0000**	0.2616	0.0904	0.2520	**0.0000**	**0.0001**
EL	**0.0000**	**0.0007**	**0.0000**	**0.0001**	0.1772	0.7866	0.0520	0.6074	**0.0000**	**0.0032**	0.1602	0.2968
KPR	**0.0003**	**0.0000**	0.6068	**0.0081**	0.2317	**0.0000**	**0.0000**	0.3476	**0.0000**	0.0470	0.0470	**0.0006**
ED	**0.0000**	**0.0000**	**0.0000**	**0.0000**	0.0132	0.6861	**0.0016**	0.0217	**0.0001**	**0.0000**	**0.0000**	**0.0000**
δ^13^C	0.2064	0.8041	**0.0000**	0.9565	**0.0038**	0.9202	0.5886	0.9262	**0.0068**	0.9578	**0.0000**	**0.0000**
P_INTsolid	**0.0036**	0.0279	0.0257	0.0245	0.0439	0.3699	0.1292	0.3228	0.5501	**0.0003**	0.1722	**0.0001**
P_INTspot	**0.0000**	**0.0000**	**0.0010**	**0.0004**	**0.0003**	0.1783	0.0145	0.5354	0.8654	0.0148	0.4793	**0.0067**
P_EXTsolid	**0.0056**	0.0317	**0.0105**	0.0226	0.3408	0.3981	0.4552	0.3838	0.2458	**0.0024**	0.0579	**0.0004**
P_EXTspot	**0.0000**	**0.0000**	**0.0000**	**0.0000**	0.0764	0.9898	0.2556	0.1330	0.3313	**0.0039**	0.0243	0.0364
M_DENsolid	0.4075	0.2311	0.1016	0.4109	**0.0000**	0.3399	**0.0000**	0.2424	**0.0000**	0.4847	**0.0000**	0.2620
M_DENmarg	0.0630	0.2555	**0.0052**	0.1073	0.1892	0.2307	0.1698	0.0803	0.3113	0.4326	0.7582	0.6461

Note

Each *p*‐value denotes the significance of the difference in phenotype least square means between two groups that differ in level of Garden (H/L Site), Continent (Mex/SA), or Population (H/L Pop), while other levels are held constant. Bold *p*‐values meet statistical significance after Bonferroni correction for multiple comparisons (α = 0.05/4 = 0.0125).

Contrary to expectations, agronomic plant fitness was higher in the High Site than the Low Site (*t*‐ratio = 7.039, *p *< 0.0001). Plant biomass and yield trait values were predicted to be higher in the Low Site. For comparison, plant height (PH, Figure 4f) is elevated in the Low Site relative to the High Site (*t*‐ratio = −7.543, *p *< 0.0001). This fitness difference is likely due to the impact of virus damage in the Low Site significantly depressing ear weight (EW, Figure 4g) values (*t*‐ratio = 8.417, *p *< 0.0001), which are a component in the adjusted agronomic fitness variable.

Flowering took longer in the High Site (days to anthesis, DTA, Figure 4c, *t*‐ratio = 69.321, *p *< 0.0001; days to silking, DTS, Figure 4d, *t*‐ratio = 71.525, *p *< 0.0001). Though all populations showed similar patterns, SA took longer to flower than Mex (days to anthesis, *t*‐ratio = −3.340, *p *< 0.001; days to silking, *t*‐ratio = −4.008, *p *< 0.0001), and Low took longer than High (days to anthesis, *t*‐ratio = −7.503, *p *< 0.0001; days to silking, *t*‐ratio = −6.908, *p *< 0.0001). Anthesis‐silking interval (ASI, Figure 4e) was lower in the High Site than in the Low Site (*t*‐ratio = −3.404, *p *< 0.001).

*δ* ^13^C (Figure 4h) values were more negative in the High Site (*t*‐ratio = −2.569, *p *< 0.01) and in the High populations (*t*‐ratio = −5.450, *p *< 0.0001). While SA Low, SA High, and Mex High did not vary significantly for *δ* ^13^C, SA High showed a distinct pattern of high *δ* ^13^C in the Low Site, similar to both Low populations, and low *δ* ^13^C in the High Site, similar to Mex High.

The High Site was characterized by increased values of solid‐pattern anthocyanin intensity (P_INTsolid, Figure 4i, *t*‐ratio = 4.769, *p *< 0.0001) and extent (P_EXTsolid, Figure 4j, *t*‐ratio = 4.839, *p *< 0.0001). High populations had higher intensity (*t*‐ratio = 3.983, *p *< 0.0001) and extent (*t*‐ratio = 4.044, *p *< 0.0001) than Low populations. Mex High and Mex Low had more similar values of anthocyanin intensity and extent, but SA High had much higher values of each than SA Low (intensity, *t*‐ratio = 4.558, *p *< 0.0001; extent, *t*‐ratio = 3.970, *p *< 0.0001).

None of the populations varied significantly in leaf sheath macrohair density (M_DENsolid, Figure 4k) between sites. The only significant differences were that Mex High had greater macrohair density than Mex Low (*t*‐ratio = 8.738, *p *< 0.0001) and SA High (*t*‐ratio = 8.961, *p *< 0.0001).

#### 3.3.3 *Q*_ST_/*F*_ST_ comparisons

**FIGURE 5:**
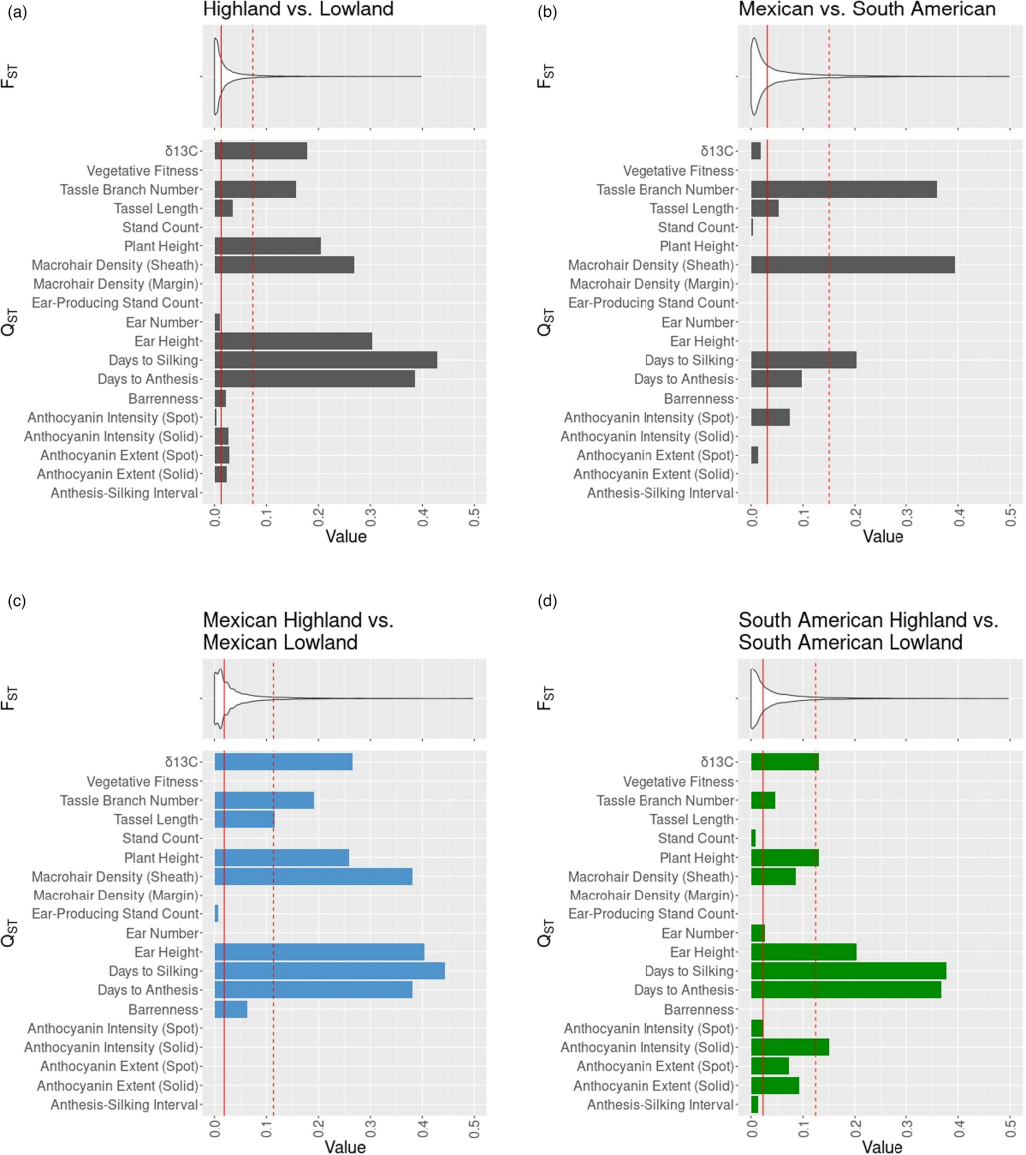
*F*_ST_ and *Q*_ST_ values between four sets of populations. Solid red lines indicate mean *F*_ST_ and dashed red lines indicate two standard deviations from the mean. (a) Highland vs. Lowland. (b) Mexican vs. South American. (c) Mexican Highland vs. Mexican Lowland. (d) South American Highland vs. South American Lowland

*Q*_ST_ values for quantitative traits were plotted against the distribution of *F*_ST_ values (Figure 5).

Four sets of comparisons were carried out: High vs. Low (Figure 5a), Mex vs. SA (Figure 5b), Mex High vs. Mex Low (Figure 5c), and SA High vs. SA Low (Figure 5d). *Q*_ST_ values more than two standard deviations above the mean *F*_ST_ were considered significantly high. In all three elevational comparisons, plant height, ear height, days to silking, and *δ* ^13^C had significantly high *Q*_ST_. *Q*_ST_ values for tassel length, tassel branch number, solid‐pattern anthocyanin pigmentation intensity, and leaf sheath macrohair density also met this threshold of significance in one or two of the elevational contrasts. In the Mex vs. SA contrast, only three traits (tassel branch number, leaf sheath macrohair density, and days to silking) had significantly high *Q*_ST_ values.

#### 3.3.4 Fitness and environmental distance

**FIGURE 6:**
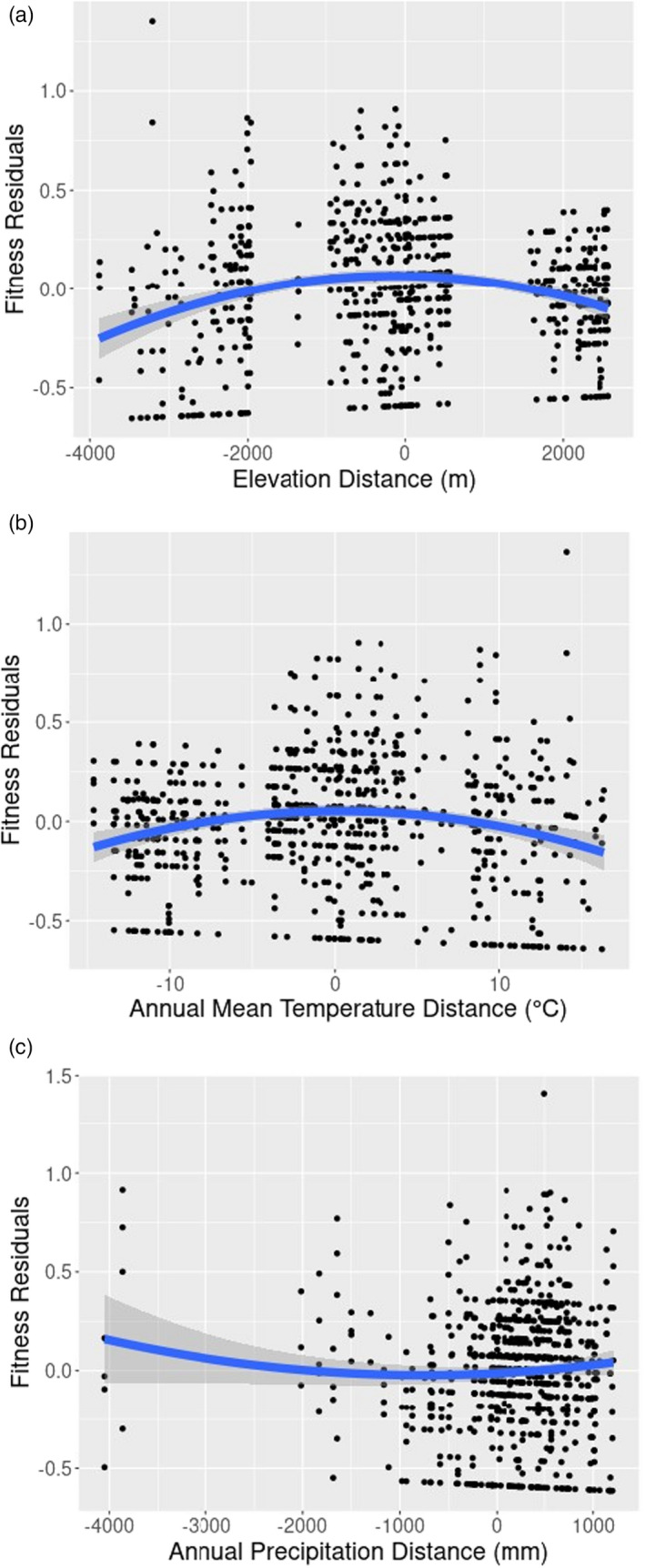
Residual plots of fitness (FITplantveg) regressed with difference in environmental variable values between accession collection sites and common garden sites. Blue lines show the fit of a quadratic model to the residuals, and grey regions indicate the 95% confidence interval. (a) Elevational distance. (b) Annual mean temperature distance. (c) Annual precipitation distance

The residuals of linear regressions of fitness values to environmental distances (differences in environmental values between the common garden site and the accession origin site) were tested for goodness‐of‐fit to a quadratic model (Figure 6). Residuals fitting a downward‐opening parabolic trend with a vertex near *y* = 0 indicate decreasing fitness with increasing environmental distance. Variance in the fitness residuals explained by the quadratic model is quantified as *R* ^2^, and *p*‐values denote the probability that *t*‐values of the quadratic coefficient are equal to zero (no quadratic relationship between fitness residual value and environmental distance).

This model finds that fitness decreases with greater distance of elevation (*p *< 0.0005) and annual mean temperature (*p *< 0.0005), but no significant trend is found for annual precipitation (*p* = 0.07). Significant decreases in fitness with increasing environmental distance were also found for the environmental variables isothermality (*p *< 0.005), max temperature of the warmest month (*p *< 0.0005), min temperature of the coldest month (*p *< 0.05), and mean temperature of the wettest (*p *< 0.0005), driest (*p *< 0.005), and warmest (*p *< 0.0005) quarters. Conversely, significant increases in fitness were found with greater distance in precipitation of the driest month (*p *< 0.0005) and quarter (*p *< 0.0005). All twenty plots are available in Figure S6, and summary statistics of the fit of the quadratic model are available in Table S2. Correlation and clustering of the 19 bioclim variables and elevation are available in Figure S7.

## 4 DISCUSSION

### 4.1 Local adaptation and plasticity

Landraces may respond to environmental changes (including climate change and dispersal to new environments) in up to four ways: plasticity, evolution, gene flow, or extinction (Mercer & Perales, 2010). The failure of an organism to plastically adapt to all available environments promotes the evolution of adaptations to a particular environment at the expense of others, a compromise known as an adaptive trade‐off. When a population evolves traits that give it a home‐site advantage over non‐native populations, that population exhibits local adaptation (Kawecki & Ebert, 2004).

All four of our elevational/continental landrace populations differ in fitness component values between our highland and lowland Mexican field sites. We observed that populations exhibited reciprocal home‐site advantage in several ways. Populations grown at sites near their native elevation had higher agronomic and vegetative fitness, stand count, ear‐producing stand count, ear weight, ear diameter, and lower barrenness than populations foreign to that site's elevation, as indicated by crossing reaction norms between populations from the same continent. Other traits showed evidence of home‐site advantage for populations from one continent, but not the other, indicating that highland and lowland populations from different continents have different adaptive strategies.

In several cases, populations also fit the “Home vs. Away” model of local adaptation, in which a population has greater fitness in the site corresponding to its native home environment than in the away site, regardless of the fitness of other populations. The Mexican Highland and Lowland populations demonstrated this pattern most clearly with ear‐producing stand count (Figure S5d). Though their reaction norms do not cross, both populations had higher fitness in their home sites. We might consider that, when populations meet the requirements for both models of local adaptation, there is a particularly strong case for local adaptation.

Several traits showed strong environmental effects but minimal *G*×*E*. All populations responded similarly to site effects for several traits, including days to anthesis, days to silking, plant height, and to lesser extents, spot‐pattern pigment intensity and extent. These results are in alignment with expectations of depressed maize plant height and prolonged maturation process due to highland conditions (Hufford et al., 2013; Mercer & Perales, 2018).

We note that our reciprocal transplant design is not fully reciprocal in that common garden sites in South American locales were not utilized. Though we may expect to see South American populations exhibiting higher fitness than Mexican populations in such locales, this is currently speculative.

### 4.2 Highland adaptation traits

#### 4.2.1 Anthocyanin pigmentation and macrohair density

We find that the intensity and extent of anthocyanin pigmentation on leaf sheaths is elevated in the highland garden site. In general, highland populations have greater overall pigmentation intensity and extent, though all populations demonstrate similar plastic effects in response to environment. *Q*_ST_ of solid‐pattern anthocyanin intensity is significantly high between South American Highland and South American Lowland, signifying selective divergence in this trait between these two populations (Merilä & Crnokrak, 2001). In contrast, Mexican Highland and Mexican Lowland have low *Q*_ST_ for this trait. The correlations between both patterns of anthocyanin and fitness appear to become more positive with increasing elevation, though solid anthocyanin pigmentation has a somewhat more positive correlation with fitness than does anthocyanin spots.

Leaf sheath macrohair density was largely non‐plastic to the environmental variation present in this study. Leaf sheath macrohair density is much greater in Mexican Highland maize than in the other populations, and this difference is greater than expected given neutral genetic loci. Introgression at macrohair‐density QTLs from *mexicana* into Highland Mexican maize (Lauter et al., 2004), followed by selection for that phenotype in the highland environment, would account for this pattern.

#### 4.2.2 Flowering time and plant maturation

Flowering time is a complex, multigenic trait that plays a crucial role in elevation adaptation (Buckler et al., 2009; Navarro et al., 2017; Wang et al., 2021). Fast flowering time is a critical component of adaptation to cold highland conditions, as plants must complete their life cycle in a narrower window of hospitable weather. In accordance with these expectations, highland populations matured more quickly than lowland populations, and this difference was more pronounced in the highland site. At the same time, maize plants from all four populations had longer flowering time in the highland site, due to the slower accumulation of growing degree days. Strong signals of *Q*_ST_ > *F*_ST_ support strong divergent selection between highland and lowland populations for flowering time.

Positive values of ASI indicate pollen release before silks are developed and receptive, which can lead to incomplete pollination and reduced yield. Positive values of ASI negatively correlate with yield (Mercer & Perales, 2018), but slightly negative values of ASI are likely less detrimental, as silks can remain receptive for several days, and a single plant that is shedding pollen early can pollinate many plants. For this reason, high values of ASI are generally regarded as an indicator of stress (Mercer et al., 2014). All four populations showed slightly higher ASI in the lowland site (though only South American Highland varied significantly). ASI reaction norms for Mexican populations are roughly parallel, while the South American reaction norms cross. This may be because ASI is more associated with local adaptation strategies of South American populations, or it may be that ASI is sensitive to the compounding stress of trans‐elevational and trans‐continental transplantation (Mittler, 2006). Both South American populations have ASI values resembling Mexican populations of the same elevation when grown at their native elevation, and then deviate more strongly when grown at the alternative elevation.

#### 4.2.3 Plant morphology and architecture

In maize, height is a polygenic trait with broad‐ranging fitness consequences (Lin et al., 1995). Lowland populations are taller than Highland populations, and this difference is greater than expected given neutral genetic markers. Maize plant height is both highly heritable (Peiffer et al., 2014) and highly plastic to environment; all populations were shorter when grown in the highland site, reflecting the environmental effect of colder temperatures at the highland site.

Optimal tassel size requires a tassel small enough for minimization of shading effects on the upper leaves yet large enough for sufficient pollen production (Mickelson et al., 2002), though the adaptive significance of tassel morphology is not well‐known. Our data show that Lowland populations have more branches than Highland populations (as observed by Eagles & Lothrop, 1994) and that South American populations have more branches than Mexican populations. Though tassel lengths of Highland populations were largely non‐plastic, Lowland populations experienced a significant reduction in tassel length when grown in the highland garden.

#### 4.2.4 Water use efficiency and δ^13^C

In C_4_ plants like maize, there is a negative correlation between WUE and *δ* ^13^C (Ellsworth & Cousins, 2016). Individuals with higher/less negative *δ* ^13^C scores have higher ratios of ^13^C:^12^C, meaning that they discriminate less effectively against ^13^C. Though the precise mechanism underlying this relationship is unclear, Avramova et al. (2019) found a region on Chromosome 7 which influences *δ* ^13^C, WUE, and sensitivity to drought through reduced abscisic acid and modified stomatal behavior. Because precipitation decreases with increasing elevation in Mexico and South America, higher WUE may play a role in highland adaptation.

Both Lowland populations show consistently high *δ* ^13^C, indicating low WUE. The Mexican Highland population had consistently lower *δ* ^13^C at both sites, indicating higher WUE. This finding is in accord with other published studies that detail the various drought‐adapted landraces of the Mexican highlands (Eagles & Lothrop, 1994; Hayano‐Kanashiro et al., 2009). In both Mexican Highland/Mexican Lowland and South American Highland/South American Lowland comparisons, *Q*_ST_ > *F*_ST_, indicating differential selection on WUE between highland and lowland populations on both continents. South American Highland maize, like Mexican Highland maize, had high WUE in the highland site, but WUE dropped significantly in the lowland site. This distinct drop in WUE seen in South American Highland maize may be the result of accumulated stress from being outside its native elevation and continent, though similar extreme drops in values of other fitness‐relevant traits in the South American Highland population are not observed.

Leaf sheath macrohair density is negatively correlated with *δ* ^13^C in both common garden sites, suggesting a role in WUE. It has been reported that macrohairs reduce water loss through transpiration by creating an air boundary layer around the plant (Chalker‐Scott, 1999; Schuepp, 1993). In accordance with this explanation, leaf sheath margin macrohair density had no such correlations with −*δ* ^13^C, as it does not produce such a boundary layer around the leaf sheath.

### 4.3 Population structure

For our provisional formulation of four maize landrace populations divided by continent and elevation, populations are more genetically similar to the corresponding population from the same continent. This is demonstrated by the genetic PCA, in which PC1 most clearly distinguishes Mexican from South American landraces. The patterns observed in this PCA are congruent with the genetic PCA by Kistler et al. (2018) in a diverse array of maize landrace and teosinte accessions from across the Americas, though their study divided accessions into population groups with a model‐based clustering algorithm.

sNMF offers a closer look at population structure of maize landraces from across the Americas. Three primary clusters emerge: A north‐western Mexican highland population, a South American highland population, and a pan‐American lowland population. If four clusters are permitted, the pan‐American lowland population splits into Mexican lowland and South American lowland. This same behavior is observed in the STRUCTURE analysis of 94 maize landraces from across North and South America by Takuno et al. (2015). Genetic admixture between these four clusters is primarily between adjacent populations: Mex High with Mex Low, Mex Low with SA Low, and SA Low with SA High. This pattern is consistent with neutral population genetic processes such as drift during range expansion and ongoing gene flow with neighboring populations. There are two possible reasons why there is not stronger genetic structure between Mexican and South American lowland landraces. First, this genetic structure may be preserved from the original southward dispersal of maize from the center of domestication to the secondary improvement center through the northern lowlands. The second potential scenario is ongoing gene flow across Central America sometime after the first wave of dispersal of maize into South America, either at low consistent levels or as part of a second wave of dispersal (Kistler et al., 2018, 2020).

A minority of SA High individuals show significant gene flow from Mex High. Recent work by Wang et al. (2021) using high‐depth whole‐genome resequencing data found that a significant percentage (about 10.7%) of highland‐adaptive SNPs in the Andean highland population are shared with Mesoamerican populations. This number is higher than previously reported (Takuno et al., 2015) and reveals a potential (but not predominant) role of trans‐regional migration of highland‐adapted alleles as part of Andean highland adaptation. Highland adaptation in the Andes appears to have been largely independent.

Though populations are more genetically similar to the corresponding population from the same continent, they are phenotypically more similar to the populations from the same elevation. While genetic population structure is largely shaped by demographic effects of drift during dispersal, phenotypes and phenotypic plasticity show evidence of being shaped by elevational adaptation. This is apparent for the majority of traits’ reaction norms between common garden sites, as well as the generally higher *Q*_ST_ between highland and lowland populations than that observed between Mexican and South American populations. We note, however, that our *Q*_ST_/*F*_ST_ must be interpreted with caution, as our four predefined populations are unlikely to meet the formal definition of populations since gene flow is limited between some landraces of the same population (Cubry et al., 2017).

### 4.4 Asymmetrical patterns of local adaptation

Mercer et al. (2008) found that highland populations suffer a greater reduction in fitness in lowland conditions than lowland populations do in highland conditions. They describe this pattern as asymmetrical local adaptation. Our data do not fully replicate this finding. Our agronomic fitness data approach this pattern, with relatively stable lowland fitness and more variable highland fitness, but vegetative fitness shows an opposite asymmetry with more variable lowland populations and more stable highland populations. As Mercer and colleagues focused on agronomic fitness, these results are in alignment. Any asymmetry of local adaptation found here may be sensitive to yearly fluctuations in *G*×*E* interactions at a site (Mercer & Perales, 2018). Transient biotic and abiotic stress pressures can significantly shape the interactions between a population and its environment, as we observed in the phenotypic differences between plants grown in the Low Site in the virus‐stressed 2016 environment versus the 2017 environment (Figure S3a). Multi‐year experiments may find that some environments are more stable, while others fluctuate between hospitability and inhospitability. Further studies would be required (and are recommended) to see whether patterns of asymmetry break down or are retained over time and how environmental stability affects local adaptation dynamics across elevational gradients.

### 4.5 Selective forces in maize evolution

Agroecosystems exert multiple and at times conflicting selective pressures on maize populations. Fitness is defined as (or approximated by, Savolainen et al., 2013) an organism's ability to survive and reproduce successfully in a particular environment. Fit maize plants must survive the myriad forces at work in the field (due to climate, elevation, soil type and quality, pest and weed pressure, as well as farmer‐mediated modifications to the land, such as tilling, irrigation, fertilizer, and crop rotation) to germinate, mature, develop numerous healthy seeds, and resist post‐harvest spoilage and loss. Furthermore, fit maize plants must also satisfy the desires of farmers to such a degree that the farmers will be convinced to replant the seed line in subsequent seasons. In fact, farmers more commonly report consciously selecting for culinary traits than for yield or environmental adaptations (Bellon et al., 2003). While maize populations continually evolve in response to competing selective pressures, agronomic practices and consumption patterns also evolve to maximize yield, minimize required inputs, and produce seed with desired grain type.

Though highland‐adapted landraces in Mexico and South America share phenotypic similarities, their adaptive strategies are not identical. This is evinced by highly divergent reaction norms between Mexico and South America for a few traits, notably *δ* ^13^C. Differences in highland adaptation between Mexican and South American maize may be due to drift incurred during the dispersal of landraces into and across South America, the unique selective challenges imparted by specific local highland regions, or likely a combination of both.

The diversity and complexity of selective forces at work in the maize landrace agroecosystem may impede detection of patterns of adaptation to abiotic clines like elevation, which may explain why the common garden experiment by Orozco‐Ramírez et al. (2014) failed to identify environmental adaptation as a leading factor in landrace distribution, and why the analyses of Dyer and Lopez‐Feldman (2013) found that altitude did not cleanly explain seed management practices. The clear patterns of adaptation to elevation found in this reciprocal transplant experiment and to other bioclimatic variables in the environmental distance regression analysis are perhaps more striking when considering the complicating and significant force of anthropogenic (or “artificial”) selection.

Additionally, the common garden sites were maintained at similar modern agronomic conditions (irrigation and pesticide/insecticide/fungicide inputs). This is in contrast to the diverse traditional agronomic practices utilized at smallholder farms across Mexico in which landrace diversity is maintained. This disparity between native habitat conditions and experimental common garden conditions may have reduced the observable signal of local adaptation to mitigated selection pressures. For example, common garden sites were well‐irrigated, releasing crops from reliance on precipitation events, which may explain why plant fitness decreased relatively little across most precipitation‐related bioclimatic variables in the environmental distance regression analysis. However, other environmental pressures (temperature, ultraviolet solar radiation, atmospheric pressure, etc.) are less likely to have been affected.

## 5 CONCLUSIONS

These results demonstrate that maize landraces from across the Americas are locally adapted to elevation and temperature. Landraces adapted to diverse environmental conditions are an invaluable resource for breeding efforts that rely on fewer costly and ecologically harmful inputs (Dwivedi et al., 2016). The myriad forces that influence the *in situ* conservation status of landraces are complex and dynamic, though locally adapted and evolving populations are more resilient and less likely to be supplanted by modern varieties (Perales et al., 2003). The importance of landraces as an agronomic resource is likely to increase due to growing global food demands, the proliferation of modern inbred lines, and the effects of global climate change, which will likely alter the conditions of many corn‐producing regions substantially (Bassu et al., 2014; Xu et al., 2016).

## CONFLICT OF INTEREST

The authors declare no conflict of interest.

## Supporting information

Supplementary Material

## Data Availability

Data for this study are available in Dryad at https://doi.org/10.5061/dryad.j0zpc86gg.
