# Supplementary material for: Demonstration of local adaptation in maize landraces by reciprocal transplantation
Source: Evol Appl. 2022 Apr 14;15(5):817–37. doi: 10.1111/eva.13372 (PMC9108319; doi:10.1111/eva.13372)
Supplement: Supplementary file 1 — Supplementary Material [file EVA-15-817-s001.docx]

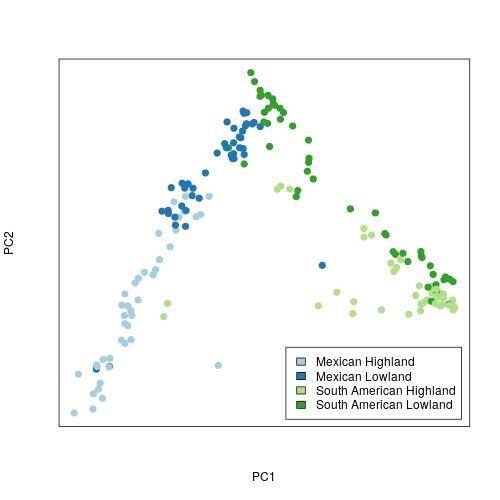


Figure S1: Population genetic structure among maize landrace populations. Principal Components Analysis of SNPs (Component 1 = 27.5%, Component 2 = 15.3%).


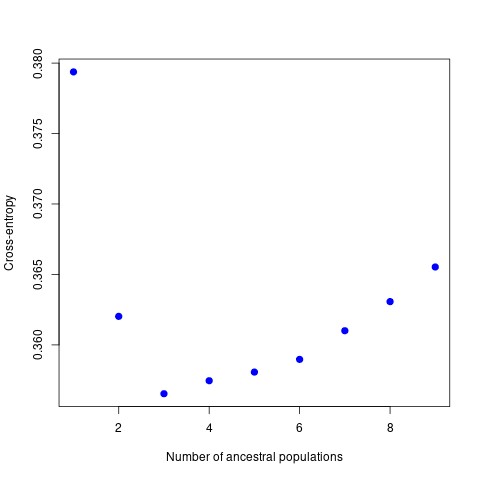


Figure S2: sNMF cross-entropy values for *K* values 1-9, finding an optimal *K* = 3.


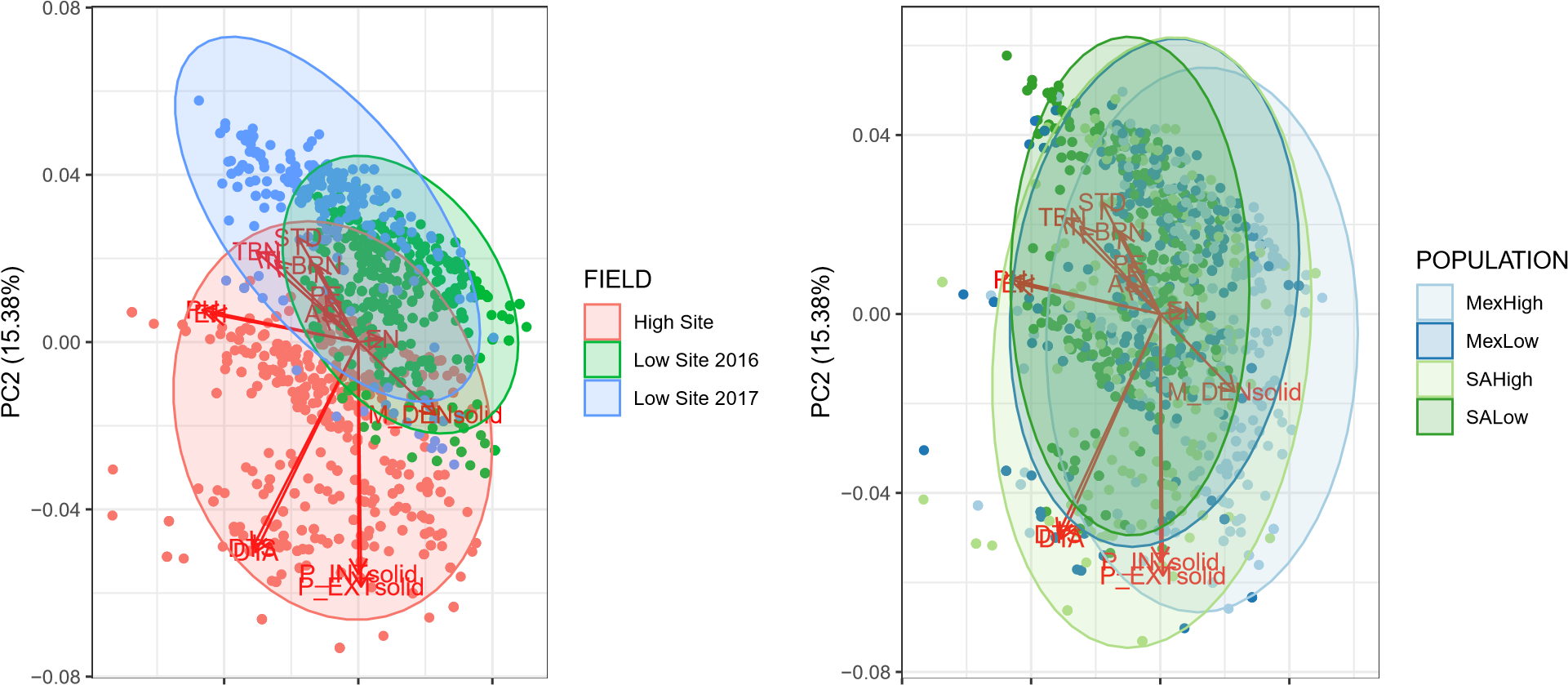


−0.05 0.00 0.05 −0.10 −0.05 0.00 0.05

PC1 (19.74%) PC1 (19.74%)

### (A) (B)

Figure S3: PCA of trait values between common garden plantings (A) and between populations

(B).


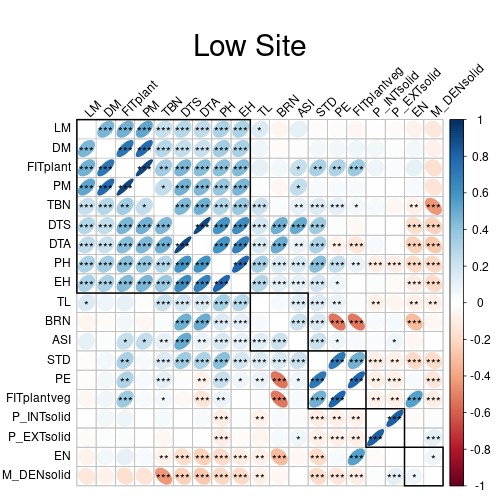

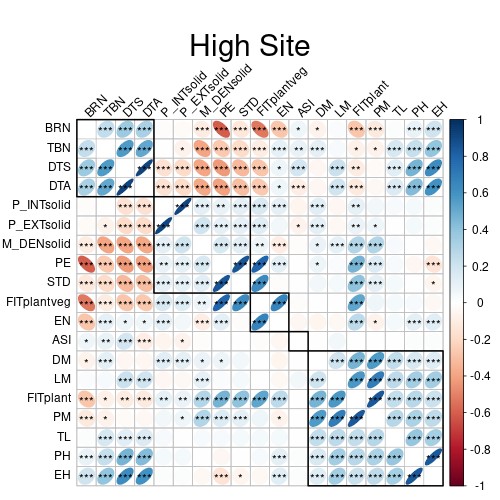


### (A) (B)

Figure S4: Pearson correlation between traits in both the lowland (A) and highland (B) common garden sites. Blue shapes indicate positive correlation, red shapes indicate negative correlation, color intensity and shape size indicate strength of correlation, and asterisks indicate statistical significance (*p*-value thresholds = 0.05, 0.01, 0.001). Squares denote the top five hierarchical clusters of traits within each common garden site.


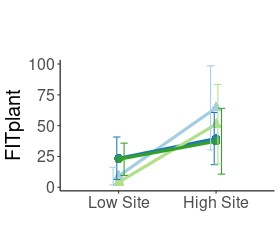

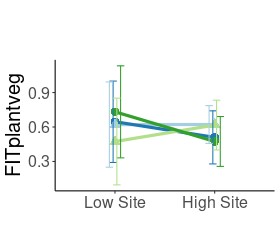

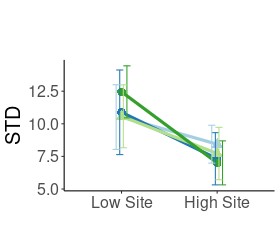

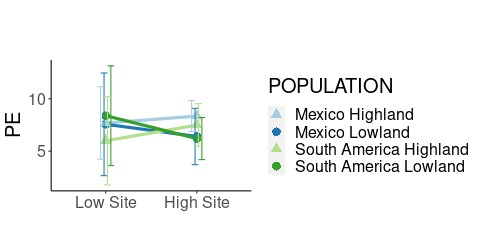


###
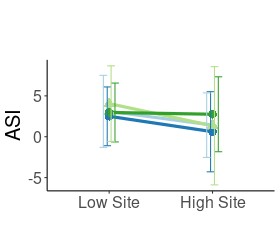
 (A) (B) (C) (D)


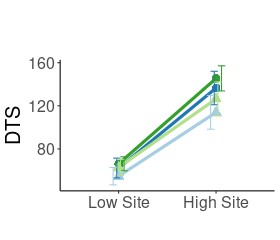


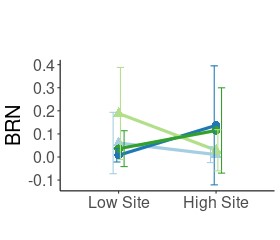

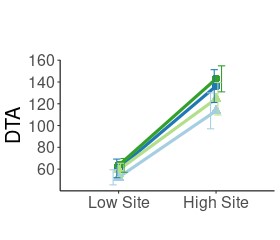


### (E) (F) (G) (H)


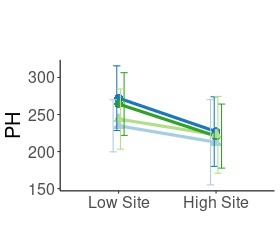

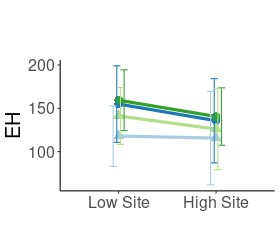

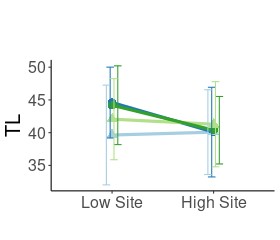

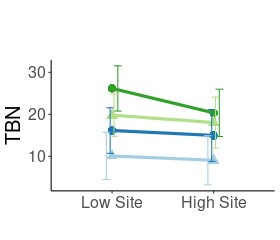


### (I) (J) (K) (L)


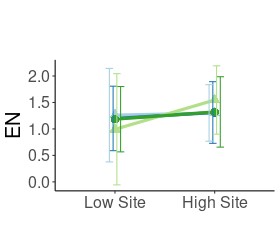

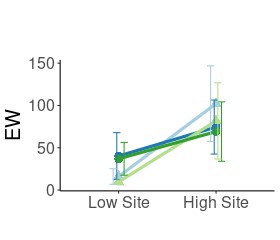

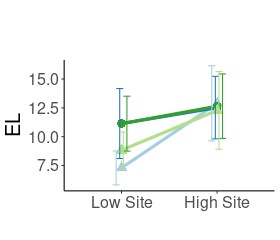

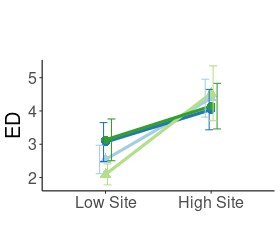


### (M) (N) (O) (P)


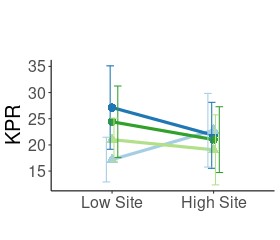

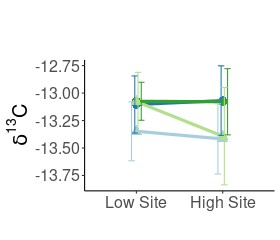

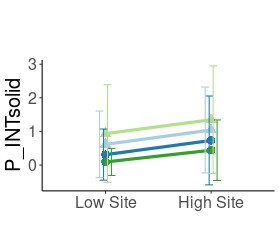

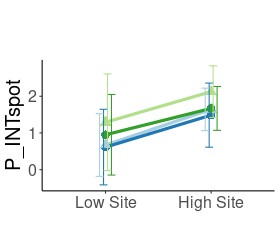


### (Q) (R) (S) (T)


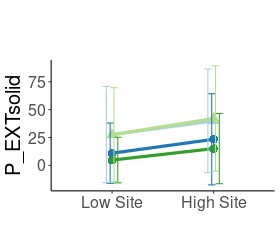

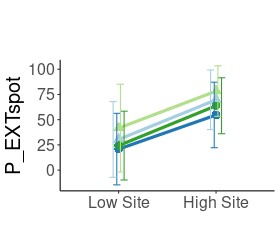

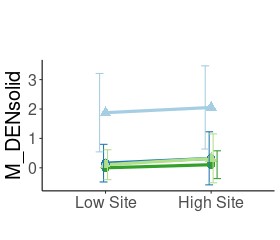

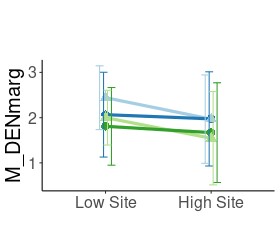


### (U) (V) (W) (X)

Figure S5: Reaction norms for all measured phenotypic traits. Error bars denote standard deviation.


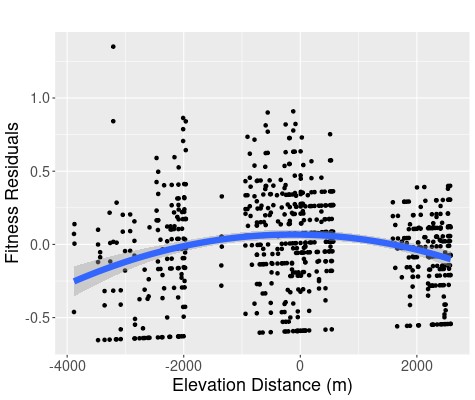

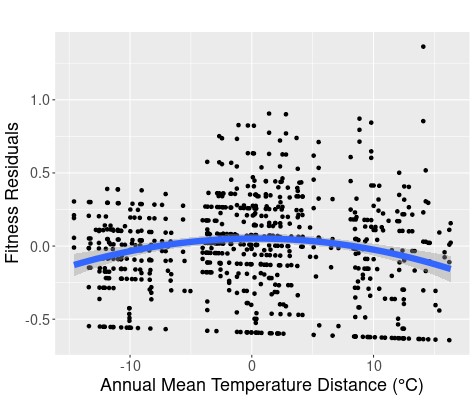

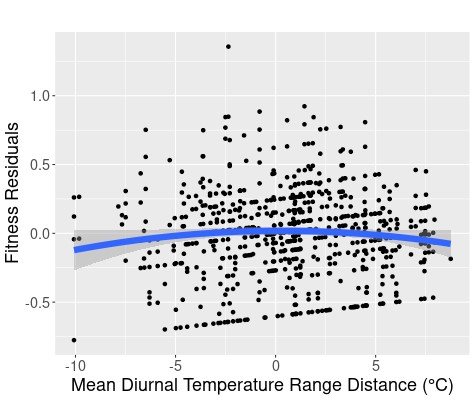


### (A) (B) (C)


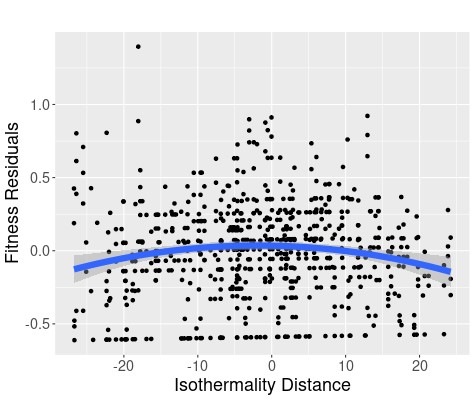

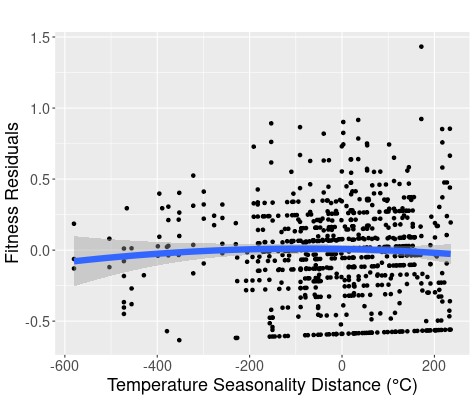

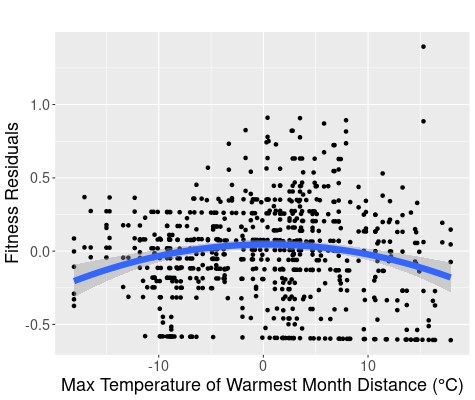


### (D) (E) (F)


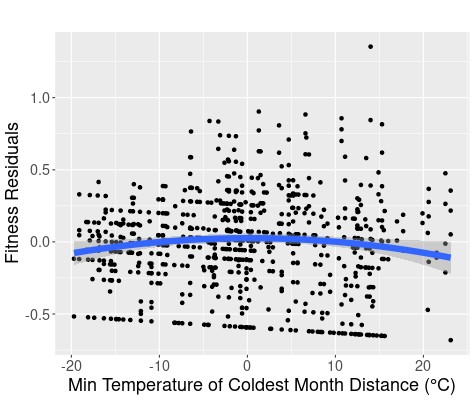

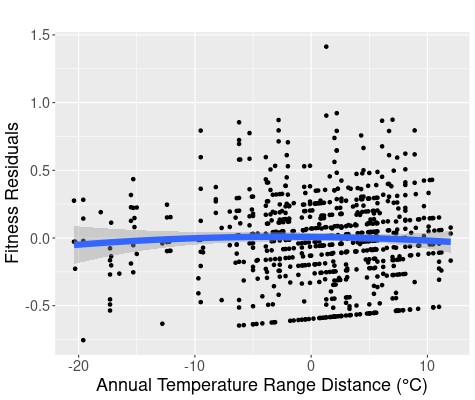


### (G) (H)


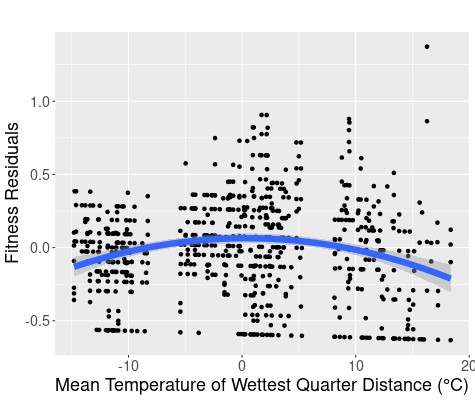

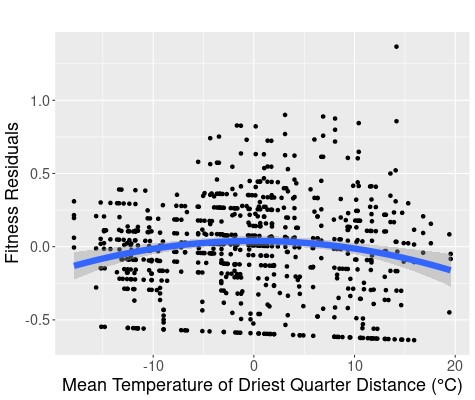


### (I) (J)

Figure S6: Residual plots of fitness (FITplantveg) regressed with difference in environmental variable values between accession collection sites and common garden sites. Blue lines show the fit of a quadratic model to the residuals.


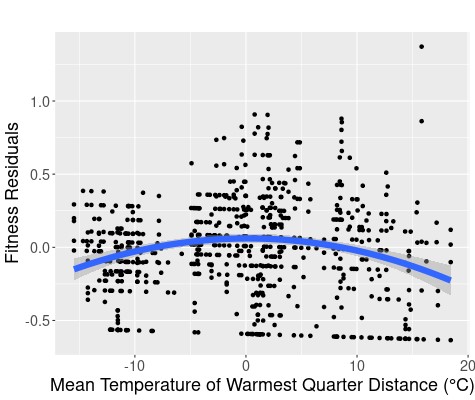

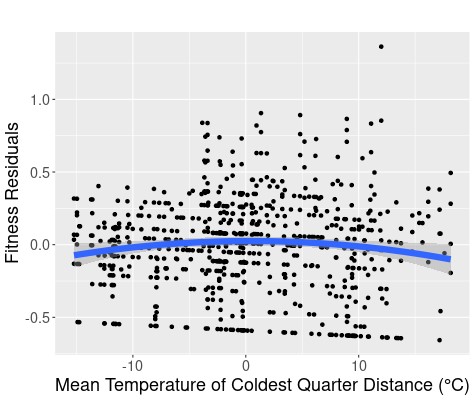

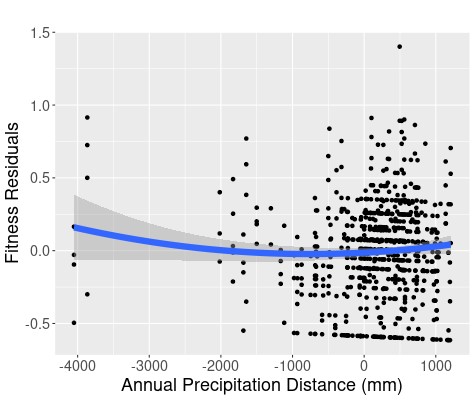


### (K) (L) (M)


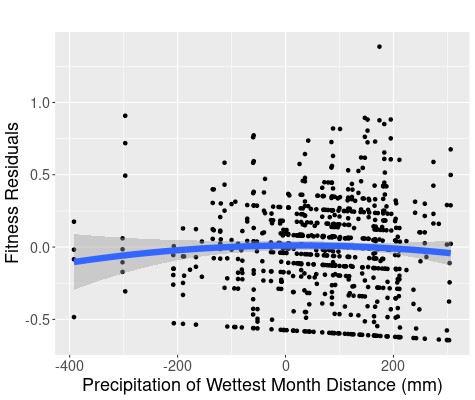

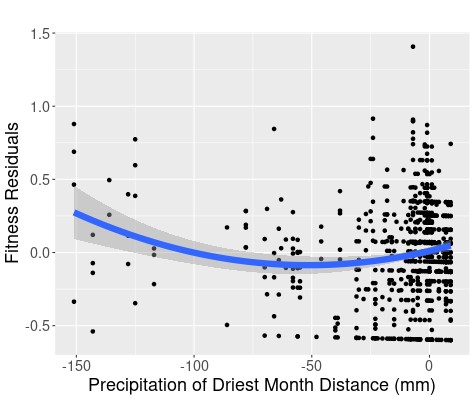

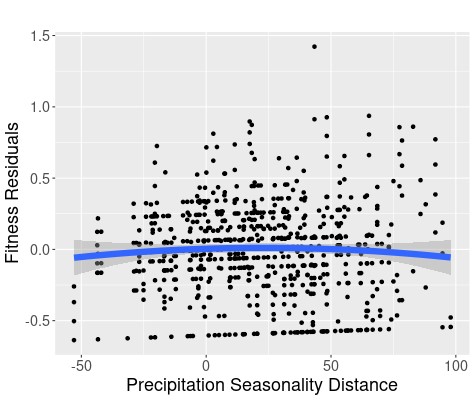


### (N) (O) (P)


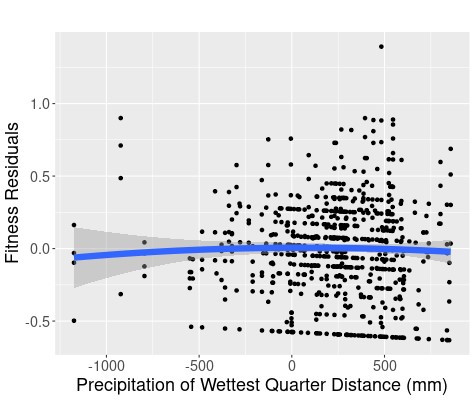

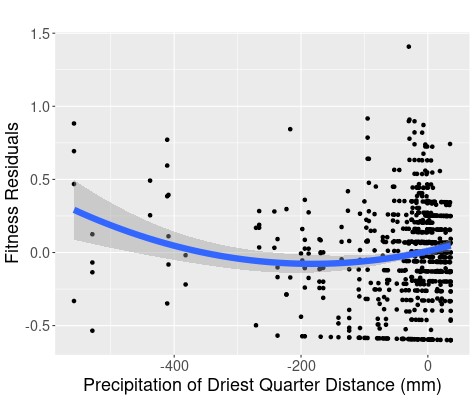


### (Q) (R)


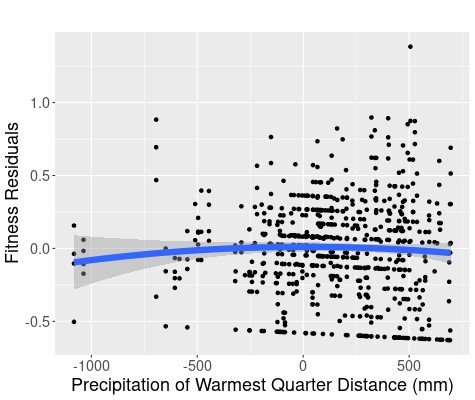

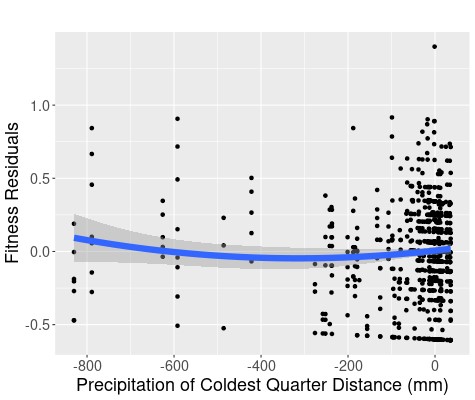


### (S) (T)

Figure S6: (Continued) Residual plots of fitness (FITplantveg) regressed with difference in environmental variable values between accession collection sites and common garden sites. Blue lines show the fit of a quadratic model to the residuals.


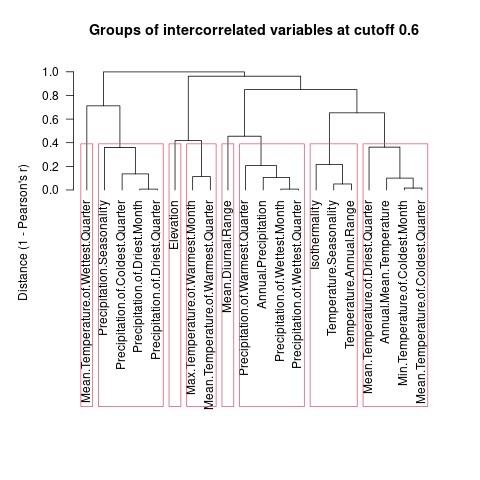


Figure S7: Hierarchical clustering of 19 bioclim variables and elevation. Red rectangles cluster variables with correlation values above 0.6.

Table S1: Passport data of the 120 landrace accessions.

| ID | Population | Pair | Long | Lat | Elevation | Race | Race Code |
| --- | --- | --- | --- | --- | --- | --- | --- |
| RIMMA0625 | Mex High | Mex 1 | -97.38 | 19.00 | 2600 | CACAHUACINTLE | Cacahuacintle |
| RIMMA0421 | Mex High | Mex 2 | -97.98 | 19.85 | 2250 | CONICO | Cónico |
| RIMMA0672 | Mex High | Mex 3 | -99.13 | 19.68 | 2256 | CHALQUENO | Chalqueño |
| CIMMYTMA-008102 | Mex High | Mex 4 | -97.24 | 19.71 | 2220 | PALOME | Palomero |
| CIMMYTMA-014017 | Mex High | Mex 5 | -101.12 | 23.13 | 2043 | NA | NA |
| CIMMYTMA-020218 | Mex High | Mex 6 | -96.27 | 16.90 | 2164 | BOLITA | Bolita |
| CIMMYTMA-026945 | Mex High | Mex 7 | -97.92 | 19.32 | 2492 | NA | NA |
| CIMMYTMA-007584 | Mex High | Mex 8 | -98.43 | 19.28 | 2261 | CONICO | Cónico |
| CIMMYTMA-027102 | Mex High | Mex 9 | -102.13 | 21.92 | 2042 | CONNOR | Cónico Norteño |
| CIMMYTMA-027432 | Mex High | Mex 10 | -92.63 | 16.75 | 2131 | NA | NA |
| CIMMYTMA-021008 | Mex High | Mex 11 | -107.76 | 29.35 | 2145 | HEMBRA | Hembra |
| CIMMYTMA-019483 | Mex High | Mex 12 | -103.48 | 22.90 | 2019 | CONNOR | Cónico Norteño |
| CIMMYTMA-001535 | Mex High | Mex 13 | -99.80 | 20.70 | 2039 | CONNOR | Cónico Norteño |
| CIMMYTMA-006081 | Mex High | Mex 14 | -97.87 | 17.19 | 2338 | CONICO | Cónico |
| CIMMYTMA-005606 | Mex High | Mex 15 | -102.27 | 19.65 | 2287 | CACAHU | Cacahuacintle |
| CIMMYTMA-021113 | Mex High | Mex 16 | -106.63 | 28.53 | 2141 | CELAYA | Celaya |
| CIMMYTMA-013920 | Mex High | Mex 17 | -97.22 | 17.45 | 2083 | NA | NA |
| CIMMYTMA-021313 | Mex High | Mex 18 | -102.17 | 21.02 | 2055 | CELAYA | Celaya |
| CIMMYTMA-016642 | Mex High | Mex 19 | -101.58 | 21.88 | 2213 | CELAYA | Celaya |
| CIMMYTMA-021154 | Mex High | Mex 20 | -107.50 | 28.55 | 2046 | CONNOR | Cónico Norteño |
| CIMMYTMA-027739 | Mex High | Mex 21 | -91.92 | 15.15 | 3201 | NA | NA |
| CIMMYTMA-013771 | Mex High | Mex 22 | -101.22 | 21.48 | 2091 | NA | NA |
| CIMMYTMA-021332 | Mex High | Mex 23 | -102.67 | 20.82 | 2017 | CELAYA | Celaya |
| CIMMYTMA-025151 | Mex High | Mex 24 | -100.14 | 20.18 | 2674 | NA | NA |
| CIMMYTMA-005613 | Mex High | Mex 25 | -101.58 | 19.50 | 2280 | MUSHIT | Mushito |
| CIMMYTMA-018374 | Mex High | Mex 26 | -99.52 | 19.97 | 2418 | NA | NA |
| CIMMYTMA-001968 | Mex High | Mex 27 | -98.68 | 18.88 | 2261 | PEPITI | Pepitilla |
| CIMMYTMA-007937 | Mex High | Mex 28 | -108.25 | 29.65 | 2141 | GORDO | Gordo |
| CIMMYTMA-013374 | Mex High | Mex 29 | -104.12 | 24.47 | 2030 | CONNOR | Cónico Norteño |
| CIMMYTMA-000554 | Mex High | Mex 30 | -101.25 | 21.02 | 2064 | CELAYA | Celaya |
| CIMMYTMA-005460 | Mex Low | Mex 1 | -100.65 | 18.35 | 245 | TUXPEN | Tuxpeño |
| CIMMYTMA-023713 | Mex Low | Mex 2 | -104.35 | 19.77 | 907 | NA | NA |
| CIMMYTMA-002335 | Mex Low | Mex 3 | -97.38 | 20.43 | 109 | TUXPEN | Tuxpeño |
| CIMMYTMA-007015 | Mex Low | Mex 4 | -105.33 | 20.32 | 655 | TUXPEN | Tuxpeño |
| CIMMYTMA-018888 | Mex Low | Mex 5 | -105.46 | 22.42 | 11 | TABLON | Tabloncillo |
| CIMMYTMA-016261 | Mex Low | Mex 6 | -101.26 | 17.56 | 52 | NA | NA |
| CIMMYTMA-000686 | Mex Low | Mex 7 | -95.44 | 18.59 | 30 | TUXPEN | Tuxpeño |
| CIMMYTMA-023180 | Mex Low | Mex 8 | -103.73 | 19.33 | 720 | NA | NA |
| CIMMYTMA-020752 | Mex Low | Mex 9 | -98.87 | 21.06 | 436 | CONICO | Cónico |
| CIMMYTMA-025027 | Mex Low | Mex 10 | -95.00 | 16.48 | 28 | ZAPCHI | Zapalote Chico |
| CIMMYTMA-000645 | Mex Low | Mex 11 | -104.56 | 29.57 | 798 | CELAYA | Celaya |
| CIMMYTMA-023628 | Mex Low | Mex 12 | -100.00 | 21.93 | 994 | CONICO | Cónico |
| CIMMYTMA-007026 | Mex Low | Mex 13 | -105.17 | 21.20 | 8 | TUXPEN | Tuxpeño |
| CIMMYTMA-025041 | Mex Low | Mex 14 | -93.72 | 16.70 | 547 | OLOTIL | Olotillo |
| CIMMYTMA-017852 | Mex Low | Mex 15 | -99.37 | 18.72 | 952 | TUXPEN | Tuxpeño |
| CIMMYTMA-007344 | Mex Low | Mex 16 | -110.37 | 29.44 | 390 | ONAVEN | Onaveño |
| CIMMYTMA-017793 | Mex Low | Mex 17 | -89.13 | 17.08 | 97 | NA | NA |
| CIMMYTMA-007076 | Mex Low | Mex 18 | -104.88 | 21.72 | 177 | BOFO | Bofo |
| CIMMYTMA-023971 | Mex Low | Mex 19 | -104.47 | 22.27 | 648 | BOFO | Bofo |
| CIMMYTMA-000449 | Mex Low | Mex 20 | -100.73 | 28.27 | 356 | TUXPEN | Tuxpeño |

Table S1: (Continued) Passport data of the 120 landrace accessions.

| ID | Population | Pair | Long | Lat | Elevation | Race | Race Code |
| --- | --- | --- | --- | --- | --- | --- | --- |
| RIMMA0409 | Mex Low | Mex 21 | -92.90 | 15.43 | 107 | TEPECINTLE | Tepecintle |
| CIMMYTMA-003266 | Mex Low | Mex 22 | -97.75 | 21.10 | 88 | TEPECI | Tepecintle |
| CIMMYTMA-025845 | Mex Low | Mex 23 | -98.38 | 21.52 | 42 | TUXPEN | Tuxpeño |
| CIMMYTMA-025289 | Mex Low | Mex 24 | -104.40 | 20.80 | 800 | NA | NA |
| CIMMYTMA-003229 | Mex Low | Mex 25 | -96.87 | 20.32 | 5 | TUXPEN | Tuxpeño |
| CIMMYTMA-029313 | Mex Low | Mex 26 | -98.27 | 20.87 | 429 | TEPECI | Tepecintle |
| CIMMYTMA-005339 | Mex Low | Mex 27 | -102.80 | 19.06 | 324 | VANDEN | Vandeño |
| CIMMYTMA-007294 | Mex Low | Mex 28 | -109.40 | 28.98 | 454 | TABPER | Tabloncillo Perla |
| CIMMYTMA-000861 | Mex Low | Mex 29 | -107.46 | 24.82 | 46 | CHAPAL | Chapalote |
| CIMMYTMA-000547 | Mex Low | Mex 30 | -99.63 | 21.45 | 562 | TUXPEN | Tuxpeño |
| CIMMYTMA-015032 | SA High | SA 1 | -65.83 | -17.55 | 2725 | NA | NA |
| CIMMYTMA-012291 | SA High | SA 2 | -74.87 | -12.63 | 3263 | NA | NA |
| CIMMYTMA-013311 | SA High | SA 3 | -79.24 | -3.62 | 2497 | MEZCLA | Mixture of Races |
| CIMMYTMA-018676 | SA High | SA 4 | -68.15 | -16.50 | 3929 | NA | NA |
| CIMMYTMA-008360 | SA High | SA 5 | -76.19 | -10.14 | 2301 | NA | NA |
| CIMMYTMA-012085 | SA High | SA 6 | -77.24 | -9.39 | 3524 | NA | NA |
| CIMMYTMA-012231 | SA High | SA 7 | -72.90 | -13.65 | 2154 | CHUNCH | Chuncho |
| CIMMYTMA-012342 | SA High | SA 8 | -78.04 | -7.80 | 3217 | SHAJAT | Shajata |
| CIMMYTMA-018242 | SA High | SA 9 | -72.21 | -13.42 | 3326 | PISCCO | Piscorunto |
| CIMMYTMA-014497 | SA High | SA 10 | -69.42 | -19.31 | 2430 | HARTAR | Harinoso Tarapaqueño |
| CIMMYTMA-018635 | SA High | SA 11 | -71.48 | -16.47 | 2495 | NA | NA |
| CIMMYTMA-016977 | SA High | SA 12 | -78.04 | 0.56 | 2484 | NA | NA |
| CIMMYTMA-008361 | SA High | SA 13 | -74.25 | -12.93 | 2655 | NA | NA |
| CIMMYTMA-018202 | SA High | SA 14 | -77.77 | -8.56 | 3412 | NA | NA |
| CIMMYTMA-014242 | SA High | SA 15 | -64.70 | -19.13 | 2400 | MOROCH | Morocho |
| CIMMYTMA-015009 | SA High | SA 16 | -65.08 | -18.18 | 2520 | AYZUMA | Ayzuma |
| CIMMYTMA-008025 | SA High | SA 17 | -65.35 | -23.20 | 2973 | NA | NA |
| CIMMYTMA-012290 | SA High | SA 18 | -73.72 | -13.52 | 2813 | NA | NA |
| CIMMYTMA-014359 | SA High | SA 19 | -65.23 | -21.37 | 2900 | AYZUMA | Ayzuma |
| CIMMYTMA-024894 | SA High | SA 20 | -78.65 | -6.55 | 2391 | NA | NA |
| CIMMYTMA-024925 | SA High | SA 21 | -75.32 | -11.90 | 3315 | SNJERO | San Jerónimo |
| CIMMYTMA-018244 | SA High | SA 22 | -76.56 | -9.41 | 3135 | NA | NA |
| CIMMYTMA-022843 | SA High | SA 23 | -73.10 | 5.40 | 2150 | SABANE | Sabanero |
| CIMMYTMA-012541 | SA High | SA 24 | -77.72 | -9.18 | 2453 | NA | NA |
| CIMMYTMA-014466 | SA High | SA 25 | -69.23 | -20.08 | 2609 | CACHCH | Calchalqui Chileno Chico |
| CIMMYTMA-021797 | SA High | SA 26 | -78.46 | -0.15 | 2890 | MISHCA | Mishca |
| CIMMYTMA-024902 | SA High | SA 27 | -71.74 | -13.95 | 3061 | NA | NA |
| CIMMYTMA-012440 | SA High | SA 28 | -79.71 | -4.64 | 2641 | NA | NA |
| CIMMYTMA-024483 | SA High | SA 29 | -65.76 | -23.53 | 3488 | PISING | Pisingallo |
| CIMMYTMA-014996 | SA High | SA 30 | -64.77 | -20.78 | 3100 | MOROCH | Morocho |
| CIMMYTMA-016874 | SA Low | SA 1 | -63.31 | -17.90 | 516 | NA | NA |
| CIMMYTMA-009022 | SA Low | SA 2 | -73.09 | -12.62 | 751 | PIRICI | Piricinco |
| CIMMYTMA-010248 | SA Low | SA 3 | -79.75 | -2.76 | 2 | NA | NA |
| CIMMYTMA-028912 | SA Low | SA 4 | -60.67 | -17.42 | 324 | CUBAAM | Cuban Yellow Flint |
| CIMMYTMA-008962 | SA Low | SA 5 | -78.42 | -9.24 | 49 | PERLA | Perla |
| CIMMYTMA-014588 | SA Low | SA 6 | -75.51 | -9.03 | 306 | NA | NA |
| CIMMYTMA-017281 | SA Low | SA 7 | -65.20 | -14.39 | 149 | NA | NA |
| CIMMYTMA-026062 | SA Low | SA 8 | -76.69 | -7.17 | 269 | NA | NA |
| CIMMYTMA-016832 | SA Low | SA 9 | -76.47 | -12.73 | 260 | NA | NA |
| CIMMYTMA-014470 | SA Low | SA 10 | -70.09 | -20.26 | 513 | HARTAR | Harinoso Tarapaqueño |

Table S1: (Continued) Passport data of the 120 landrace accessions.

| ID | Population | Pair | Long | Lat | Elevation | Race | Race Code |
| --- | --- | --- | --- | --- | --- | --- | --- |
| CIMMYTMA-014998 | SA Low | SA 11 | -60.66 | -16.78 | 300 | CUBAAM | Cuban Yellow Flint |
| CIMMYTMA-008156 | SA Low | SA 12 | -79.83 | 0.84 | 17 | MCUBAN | Mixed Cuban Yellow Flint |
| CIMMYTMA-008367 | SA Low | SA 13 | -76.95 | -12.13 | 305 | NA | NA |
| CIMMYTMA-014578 | SA Low | SA 14 | -76.47 | -8.44 | 580 | NA | NA |
| CIMMYTMA-011857 | SA Low | SA 15 | -59.59 | -18.35 | 613 | NA | NA |
| CIMMYTMA-011854 | SA Low | SA 16 | -57.83 | -18.97 | 120 | POJOSO | Pojoso Chico, Ecuador |
| CIMMYTMA-014287 | SA Low | SA 17 | -64.41 | -22.54 | 559 | CUBAAM | Cuban Yellow Flint |
| CIMMYTMA-015086 | SA Low | SA 18 | -67.37 | -14.32 | 189 | NA | NA |
| CIMMYTMA-014283 | SA Low | SA 19 | -63.67 | -21.92 | 640 | CUBAAM | Cuban Yellow Flint |
| CIMMYTMA-026029 | SA Low | SA 20 | -75.30 | -7.22 | 137 | NA | NA |
| CIMMYTMA-009025 | SA Low | SA 21 | -70.25 | -12.00 | 234 | ENANO | Enano |
| CIMMYTMA-026034 | SA Low | SA 22 | -74.90 | -10.33 | 252 | NA | NA |
| CIMMYTMA-019738 | SA Low | SA 23 | -74.92 | 5.32 | 840 | NA | NA |
| CIMMYTMA-008993 | SA Low | SA 24 | -78.73 | -8.60 | 14 | COLORA | Colorado |
| CIMMYTMA-014383 | SA Low | SA 25 | -63.29 | -20.68 | 755 | CUBAAM | Cuban Yellow Flint |
| CIMMYTMA-017379 | SA Low | SA 26 | -80.44 | -1.07 | 36 | NA | NA |
| CIMMYTMA-016810 | SA Low | SA 27 | -75.71 | -14.18 | 381 | NA | NA |
| CIMMYTMA-026035 | SA Low | SA 28 | -80.71 | -4.87 | 49 | NA | NA |
| CIMMYTMA-026714 | SA Low | SA 29 | -61.48 | -23.78 | 175 | PERLIT | Perlita |
| CIMMYTMA-014314 | SA Low | SA 30 | -63.19 | -21.44 | 600 | CUBAAM | Cuban Yellow Flint |

Table S2: Significance of quadratic fit of fitness (FITplantveg) distance residuals

|  | *R*^2^ | *SE* | *t* | *p*(*>*\|*t*\|) |
| --- | --- | --- | --- | --- |
| Elevation | 0.0443 | 0.0000 | -5.8156 | 0.0000 |
| Annual Mean Temperature | 0.0248 | 0.0016 | -4.4066 | 0.0000 |
| Mean Diurnal Range | 0.0022 | 0.0033 | -1.8691 | 0.0620 |
| Isothermality | 0.0136 | 0.0011 | -3.3886 | 0.0007 |
| Temperature Seasonality | -0.0014 | 0.0001 | -1.0355 | 0.3008 |
| Max Temperature of Warmest Month | 0.0238 | 0.0016 | -4.3261 | 0.0000 |
| Min Temperature of Coldest Month | 0.0051 | 0.0012 | -2.3432 | 0.0194 |
| Temperature Annual Range | -0.0017 | 0.0022 | -0.9036 | 0.3665 |
| Mean Temperature of Wettest Quarter | 0.0382 | 0.0014 | -5.4049 | 0.0000 |
| Mean Temperature of Driest Quarter | 0.0135 | 0.0014 | -3.3798 | 0.0008 |
| Mean Temperature of Warmest Quarter | 0.0368 | 0.0015 | -5.3107 | 0.0000 |
| Mean Temperature of Coldest Quarter | 0.0047 | 0.0016 | -2.2904 | 0.0223 |
| Annual Precipitation | 0.0018 | 0.0000 | 1.8074 | 0.0711 |
| Precipitation of Wettest Month | -0.0007 | 0.0001 | -1.2199 | 0.2229 |
| Precipitation of Driest Month | 0.0198 | 0.0010 | 3.9849 | 0.0001 |
| Precipitation Seasonality | -0.0012 | 0.0007 | -1.0785 | 0.2812 |
| Precipitation of Wettest Quarter | -0.0023 | 0.0000 | -0.6752 | 0.4998 |
| Precipitation of Driest Quarter | 0.0168 | 0.0003 | 3.7023 | 0.0002 |
| Precipitation of Warmest Quarter | -0.0009 | 0.0000 | -1.1888 | 0.2349 |
| Precipitation of Coldest Quarter | 0.0011 | 0.0002 | 1.6651 | 0.0964 |

Bold *p*-values meet statistical significance after Bonferroni correction for multiple comparisons (*α* = 0*.*05*/*20 = 0*.*0025).
